# Readout of histone methylation by Trim24 locally restricts chromatin opening by p53

**DOI:** 10.1038/s41594-023-01021-8

**Published:** 2023-06-29

**Authors:** Luke Isbel, Murat Iskar, Sevi Durdu, Joscha Weiss, Ralph S. Grand, Eric Hietter-Pfeiffer, Zuzanna Kozicka, Alicia K. Michael, Lukas Burger, Nicolas H. Thomä, Dirk Schübeler

**Affiliations:** 1 Friedrich Miescher Institute for Biomedical Research, Basel, Switzerland; 2 School of Biotechnology and Biomolecular Sciences, University of New South Wales, Sydney, New South Wales Australia; 3 Faculty of Sciences, University of Basel, Basel, Switzerland; 4 Zentrum für Molekulare Biologie der Universität Heidelberg, Heidelberg, Germany; 5 Biozentrum, University of Basel, Basel, Switzerland; 6 Swiss Institute of Bioinformatics, Basel, Switzerland

**Keywords:** Histone post-translational modifications, Epigenetics, Chromatin, Chromatin remodelling, Chromatin analysis

## Abstract

The genomic binding sites of the transcription factor (TF) and tumor suppressor p53 are unusually diverse with regard to their chromatin features, including histone modifications, raising the possibility that the local chromatin environment can contextualize p53 regulation. Here, we show that epigenetic characteristics of closed chromatin, such as DNA methylation, do not influence the binding of p53 across the genome. Instead, the ability of p53 to open chromatin and activate its target genes is locally restricted by its cofactor Trim24. Trim24 binds to both p53 and unmethylated histone 3 lysine 4 (H3K4), thereby preferentially localizing to those p53 sites that reside in closed chromatin, whereas it is deterred from accessible chromatin by H3K4 methylation. The presence of Trim24 increases cell viability upon stress and enables p53 to affect gene expression as a function of the local chromatin state. These findings link H3K4 methylation to p53 function and illustrate how specificity in chromatin can be achieved, not by TF-intrinsic sensitivity to histone modifications, but by employing chromatin-sensitive cofactors that locally modulate TF function.

## Abstract (web summary)

The presence of Trim24 increases cell viability upon stress and enables p53 to influence gene expression as a function of the local chromatin state.

## Main

TFs are DNA-binding proteins that determine distinct spatial and temporal transcriptional patterns. Beyond DNA sequence, chromatin proteins such as nucleosomes and their modifications are thought to add an additional layer of gene regulation^1,2^. However, it remains largely unclear how these modifications are ‘read out’ by TFs, which lack known domains that specifically interact with histones. However, such domains are abundant on non-DNA-binding transcriptional cofactors^3^.

The base unit of chromatin is the nucleosome, which consists of ~147 base pairs (bp) of DNA wrapped around a histone octamer. Nucleosome modifications such as methylation of H3K4 (H3K4me) or acetylation of H3 lysine 27 (H3K27ac) play a functional part in the actions of TFs, but the mechanism by which they do so remains enigmatic^4^. Modifications might directly affect TF access to DNA by altering nucleosome–DNA wrapping kinetics^5^. Alternatively, TFs may recruit cofactors that are sensitive to histone modifications. For example, many chromatin remodeler complexes possess a combination of chromatin-binding domains that modulate their activity, at least in vitro^4,6,7^. In the latter case, it seems possible that such cofactors enable TF specificity to regulate gene activity on the basis of local chromatin states.

High accessibility and the presence of euchromatic histone modifications are hallmarks of active regulatory regions, such as enhancers and promoters, and can be successfully used for the detection of such regions^8^. They coincide with the binding of TFs, suggesting a functional link^9^. However, these striking correlations with chromatin features do not demonstrate whether a TF creates open chromatin or binds as a consequence thereof. Loss-of-function studies suggest that some TFs are at least partially involved in maintaining an ‘open’ chromatin state at regulatory regions^10^. This makes it highly challenging to disentangle the contribution of ‘naive’ chromatin toward the initial engagement of a TF from the ongoing remodeling that occurs owing to TF binding at a steady state^11^. These issues can be partially circumvented by studying TF binding upon induction or ectopic expression, as this enables monitoring of the initial TF engagement and should reveal sensitivity to chromatin. Of particular interest has been the binding to regions of closed chromatin by so-called pioneer TFs to drive cell fate and differentiation^12^. In vitro, several TFs have been shown to engage nucleosomal DNA, although it remains to be determined whether this behavior occurs in vivo and whether histone modifications alter nucleosomal engagement^13,14^.

The TF and tumor suppressor p53 controls the expression of numerous genes involved in DNA repair, cell cycle arrest and cell death^15^. p53 can be rapidly induced in response to various forms of cellular stress, resulting in immediate activation of target genes^16,17^. p53 is unusual in that only a fraction of p53-bound sites display signs of open chromatin under cell culture conditions^17–20^. Thus, p53 seems to engage closed chromatin sites without creating accessibility until activated by stress-response pathways. However, it remains unknown how stable engagement in closed chromatin is achieved or indeed whether there are functional consequences of such binding.

Here, we show that a cofactor, Trim24, limits the degree to which p53 can create accessibility in closed chromatin and, furthermore, that it does so by reading out the local methylation state of H3K4. This provides a molecular link between histone methylation and p53 function, enabling locus-specific TF regulation subsequent to its initial binding.

## Results

### p53 engages closed chromatin in the mouse and human genomes

**Fig. 1:**
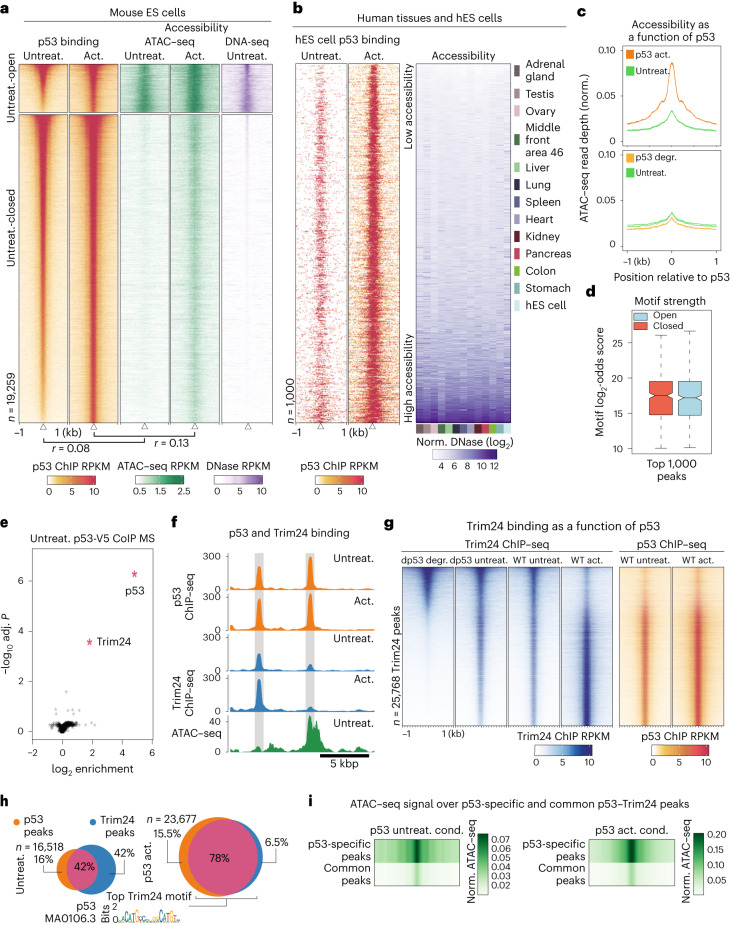
p53 binds to closed chromatin in mES cells and human tissues and recruits Trim24. **a**, p53 binds to open and closed mES cell chromatin. Binding and accessibility (middle, ATAC–seq; right, DNase-seq) increased following activation (4 h, doxorubicin 1 µM) as indicated by Pearson correlation scores (below). **b**, Human ES cell p53 ChIP–seq (left) alongside DNase-seq accessibility in human tissues and human ES cells (right). **c**, Accessibility metaprofiles (normalized ATAC–seq signal) of WT cells (top) and cells with degron-tagged p53 (bottom) after activation and degradation (dTAG13 500 nM, 4 h), respectively. **d**, Motif log_2_-odds score at top 1,000 open and closed chromatin sites, using average ChIP–seq signal from two independent replicates. Center median to first and third quartiles, whiskers to 1.5 multiplied by interquartile range. **e**, Coimmunoprecipitation with mass spectrometry of V5-tagged p53 enriched for both p53 and Trim24 proteins; red indicates proteins with Benjamini–Hochberg-adjusted two-sided eBayes *P* < 0.01. **f**, p53 and Trim24 binding (ChIP–seq) and accessibility (ATAC–seq); representative p53–Trim24-cobound sites and p53-only sites are highlighted. **g**, Heatmaps of Trim24 ChIP–seq upon p53 degradation in the dp53-degron-tagged line and upon activation in WT mES cells (left), alongside p53 ChIP–seq in WT cells (right). **h**, Overlap of p53 and Trim24 peaks under p53-uninduced (left) and p53-active (right) conditions. The top motif from the Trim24 ChIP–seq peaks is shown. **i**, ATAC–seq under uninduced (left) and active (right) conditions at common and p53-only peaks as in **h**. p53-specific peaks overlap accessible regions, whereas common Trim24-bound peaks are relatively depleted in terms of accessibility. Act., active; CoIP MS, coimmunoprecipitation with mass spectrometry; cond., condition; degr., degraded; DNase-seq, DNase I hypersensitive sites sequencing; norm., normalized; untreat., untreated.

Features of open chromatin, such as accessibility, ‘active’ histone marks and low DNA methylation, are hallmarks of sites of TF binding^9,21,22^. p53 appears to be different in this respect as it has been reported to occupy sites of closed and open chromatin^17–19^. We investigated p53 binding in mouse embryonic stem cells (mES cells), which represent a nontransformed cell type. Under uninduced cell culture conditions, p53 displayed strong binding at both open and closed chromatin loci, with stress-induced activation of p53 via doxorubicin treatment resulting in increased binding and accessibility at these sites^23^ (Fig. 1a and Supplementary Fig. 1a–f). In general, p53 motifs were highly enriched compared with others in regions that changed in accessibility, arguing for its strong relevance in stress response (Supplementary Fig. 1e). The diversity of chromatin states at p53-binding sites was also evident in human tissues and ES cells (Fig. 1b and Supplementary Fig. 2a–c). Indeed, the majority of sites that were consistently bound among various cell types were depleted in terms of their accessibility signal. In general, this behavior is in stark contrast to TFs previously shown to be chromatin insensitive, for which binding almost exclusively occurs in open chromatin, suggesting that it is more typical for TFs to create accessibility upon closed chromatin binding^24^ (Supplementary Fig. 1f).

Several reports suggest that p53 may be responsible for enabling repressive chromatin marks at binding sites^25^. To test whether p53 influences chromatin states under uninduced conditions, we tagged it to enable acute and targeted degradation. Specifically, we integrated V5 and dTAG peptides at the endogenous gene, allowing detection by the V5 epitope and dTAG-inducible degradation^26^ (Supplementary Fig. 3a,b). This *dp53* allele displayed similar expression and genomic binding compared with wild-type (WT) p53 under uninduced conditions. Upon stress activation it retained functionality, although to a lesser degree, as measured by binding, as well as the ability to create accessible chromatin and contribute to local levels of the active histone mark H3K27ac (Supplementary Fig. 3c–h). Rapid degradation of p53 did not cause any increase in accessibility at closed chromatin, arguing against a direct role for p53 in silencing (Fig. 1c and Supplementary Fig. 3e–g). These results suggest that chromatin is largely agnostic to p53 before activation and indicate the possibility that features of closed chromatin enable stable binding of p53.

### p53 binds to closed chromatin independent of DNA methylation

One feature of closed chromatin is a high level of DNA methylation. This could be relevant to p53, which has been reported to prefer some motif variants when methylated^27^. We therefore considered whether DNA methylation could account for the observed binding patterns. If so, we would expect a reduction in binding at closed chromatin sites upon removal of DNA methylation. To test this, we used chromatin immunoprecipitation followed by sequencing (ChIP–seq) to measure p53 binding in cells that lacked DNA methylation owing to genetic deletion of all three DNA methyltransferases (DNMTs)^28^. These cells were largely indistinguishable from WT cells with respect to p53 binding, with continued binding in closed chromatin (Supplementary Fig. 4a). Similarly, comparing the strongest sites located in closed and open chromatin revealed similar motif strength (Fig. 1d). We conclude that the binding of p53 to closed chromatin appears to be independent of DNA methylation.

### Trim24 localizes with p53 in the genome

We speculated that binding patterns could be explained by p53 cofactors that bind to chromatin. To identify such potential cofactors, we carried out coimmunoprecipitation with mass spectrometry on whole-cell extracts using the V5 tag on the dp53 allele. Encouragingly, p53 itself was the top enriched protein (Fig. 1e and Supplementary Fig. 5a,b), followed by the Trim24 protein with comparable enrichment. Trim24, which is a protein that possesses canonical histone-binding domains, has previously been implicated as a p53-interacting E3 ubiquitin ligase^29^. Trim24 has no DNA binding ability on its own, yet it contains PHD and bromo-domains that can interact with modified histones^30^, indicating the possibility that Trim24 links p53 binding to local chromatin.

**Extended Data Fig. 1:**
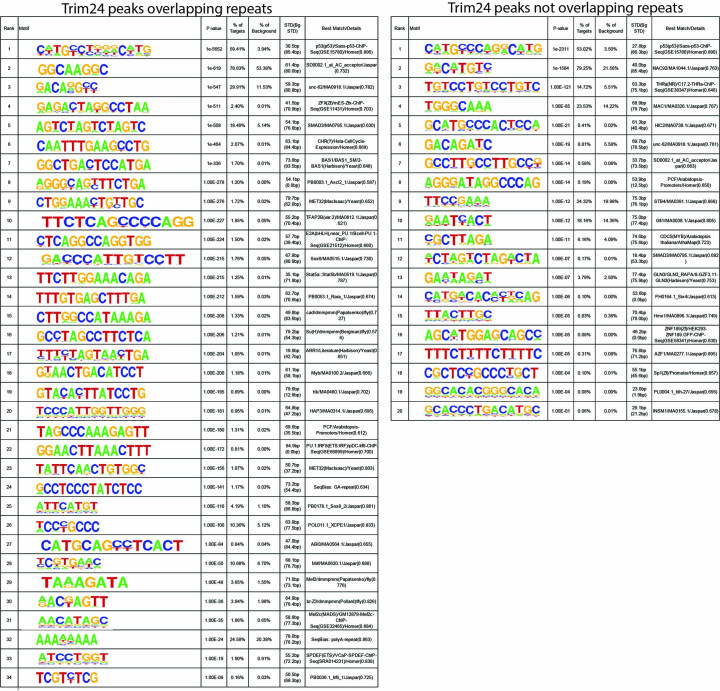
Motif enrichment at Trim24 peaks. Homer De Novo motif enrichment at Trim24 peaks either overlapping repeat masker annotated repeats (excluding simple repeats) or not overlapping repeats. The p53 motif is uniquely enriched with a high significance and presence in target sites over background in both cases.

To determine whether the observed interaction translated into co-occupancy across the genome, we carried out ChIP–seq of Trim24 under both uninduced and p53-active conditions (Fig. 1f and Supplementary Fig. 5c). The top enriched sequence at Trim24 peaks was the p53 motif, and we detected co-occupancy at the majority of peaks, with a high degree of scaling in the signal for both factors. Trim24 has been linked to individual repeat occurrences^31^. Indeed, according to our genome-wide analysis, a substantial fraction of Trim24 sites were in repeats co-occupying with p53 (Supplementary Fig. 5d). However, cobinding was not limited to repeat elements (Supplementary Fig. 5d and Extended Data Fig. 1). To determine whether Trim24 depends on p53 for binding, we measured Trim24 binding upon p53 degradation using the p53 degron line (Fig. 1g). Loss of p53 led to absence of Trim24 at p53 sites, suggesting that p53 recruits Trim24. In addition, we monitored binding upon p53 activation, which coincided with an increase in Trim24 binding at p53 sites. These data argue that Trim24 binding is influenced by local genomic levels of p53. Furthermore, purified p53 binds to the amino-terminal region of the Trim24 protein, demonstrating that the interaction is not indirect via DNA and histones on chromatin (Supplementary Fig. 5e–g).

Although co-occupancy was observed for the majority of genomic p53 sites, there was a subgroup of sites that were bound exclusively and strongly by p53 (Fig. 1f,h and Supplementary Fig. 5c). Comparing the ATAC–seq (assay for transposase-accessible chromatin using sequencing) signals at overlapping versus nonoverlapping Trim24 and p53 peaks revealed that co-occupied sites showed low to no accessibility and thus resided in closed chromatin (Fig. 1i). By contrast, sites bound by p53 alone were enriched in accessibility and resided in open chromatin. Together, these data raise the possibility that the binding of Trim24 at p53 sites depends not only on p53 but also on the local chromatin state.

### Trim24 is not required for p53 binding to closed chromatin

**Fig. 2:**
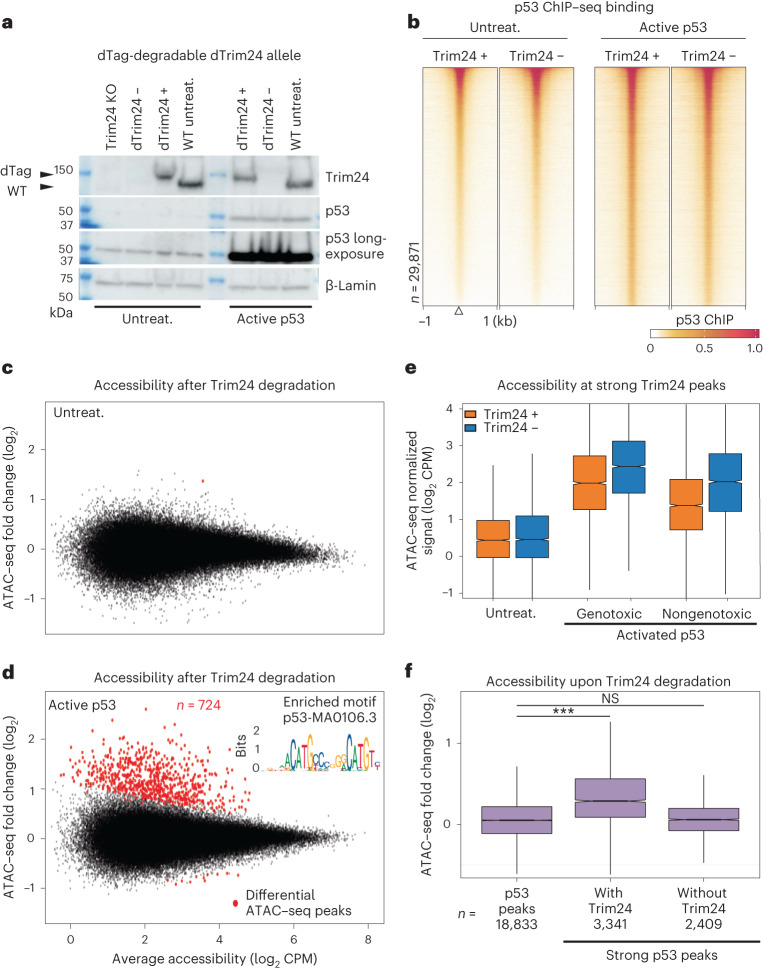
Trim24 is a negative regulator of active p53 in the genome. **a**, Tagged Trim24 can be rapidly degraded (+/− dTag) including during p53 activation (dTAG13 500 nM, doxorubicin 1 μM, 4 h). **b**, p53 ChIP–seq in Trim24-degraded mES cells at combined p53 and Trim24 peaks. **c**,**d**, MA plot of changes in accessibility (log_2_ CPM) upon degradation of Trim24, under uninduced (**c**) and active (**d**) conditions, for all mES cell ATAC–seq peaks (*n* ~165 K); red indicates edgeR FDR < 0.05. p53 motifs were enriched at differential sites under active conditions (Homer *P* < 1 × 10^−297^). **e**, ATAC–seq signal (log_2_ CPM) under uninduced, doxorubicin (genotoxic) and nutlin3a (nongenotoxic) p53-activated conditions for 4 h, with and without Trim24 loss at strong Trim24 peaks (*n* = 3,883), that is, >3.5-fold log_2_ Trim24 ChIP–seq enrichment. Center median to first and third quartile, whiskers to 1.5 multiplied by interquartile range. **f**, Increase in ATAC–seq signal at all p53 ChIP–seq peaks and strong p53 peaks with or without Trim24, that is, >3.5-fold log_2_ ChIP–seq enrichment for p53 and/or Trim24. Values represent averages from three independent experiments. Center median to first and third quartile, whiskers to 1.5 multiplied by interquartile range. Two-sided Wilcoxon rank-sum test, ***P < 0.001. KO, knockout; NS, not significant. Source data

To test whether Trim24 contributes to genomic binding of p53, we endogenously tagged *Trim24* with the V5 and dTAG sequences, which enable detection and acute degradation (Fig. 2a and Supplementary Fig. 5h). Trim24 degradation occurred rapidly but did not affect protein expression levels of p53 or decrease its genomic binding (Fig. 2a,b and Supplementary Fig. 5i). We retested this at cellular resolution using immunofluorescence microscopy; the results confirmed that Trim24 removal had minimal effects (Supplementary Fig. 6a–e). This was true under both uninduced and active p53 conditions. Only a small number of p53 sites (*n* ~150) appeared to respond to the absence of Trim24 by showing increased p53 binding (Supplementary Fig. 5i).

### Trim24 represses p53-dependent accessibility upon stress

Having found that Trim24 was not required for binding, we next considered whether it was recruited to modulate the ability of p53 to remodel chromatin. We therefore profiled accessibility in cells upon rapid loss of Trim24 under p53-uninduced conditions (Fig. 2c and Supplementary Fig. 7a,b). There were almost no changes in accessibility genome-wide, in stark contrast to the changes observed under p53-active conditions, where several hundred loci showed increased accessibility upon loss of Trim24 (Fig. 2d). These loci were highly enriched for p53 binding (Supplementary Fig. 7c,d). Only a few loci decreased in accessibility (*n* = 11). To determine whether the observed Trim24 function was specific to the cellular stress used to activate p53 and thus potentially limited to the doxorubicin-induced DNA damage, we exposed cells to two additional activation paradigms: MDM2 inhibition using nutlin3a; and DNA damage via ionizing radiation (Fig. 2e and Supplementary Fig. 7e–g). Regardless of the method used to activate p53, Trim24 loss increased accessibility at a set of strong p53 sites that responded to doxorubicin, indicating that Trim24 limits the ability of active p53 to generate local chromatin accessibility.

### Trim24 acts on chromatin in a locus-specific manner

Next, we investigated whether Trim24 affected all p53 sites or was limited to only the closed chromatin subset of p53 sites where both bind (Fig. 1h). We therefore reanalyzed accessibility at strong p53 peaks with and without Trim24 binding (Fig. 2f). This revealed an increase in accessibility at co-occupied p53–Trim24 peaks but not at p53-only peaks. Furthermore, these Trim24-dependent effects were present for up to 12 h after p53 activation (Supplementary Fig. 8a–c). Together, these data argue that the activity of Trim24 takes place at local genomic elements with specific chromatin features rather than generally within the nucleoplasm, in contrast to other direct regulators such as Mdm2 that act on total cellular p53 levels.

### Trim24 localization is modulated by H3K4 methylation

**Fig. 3:**
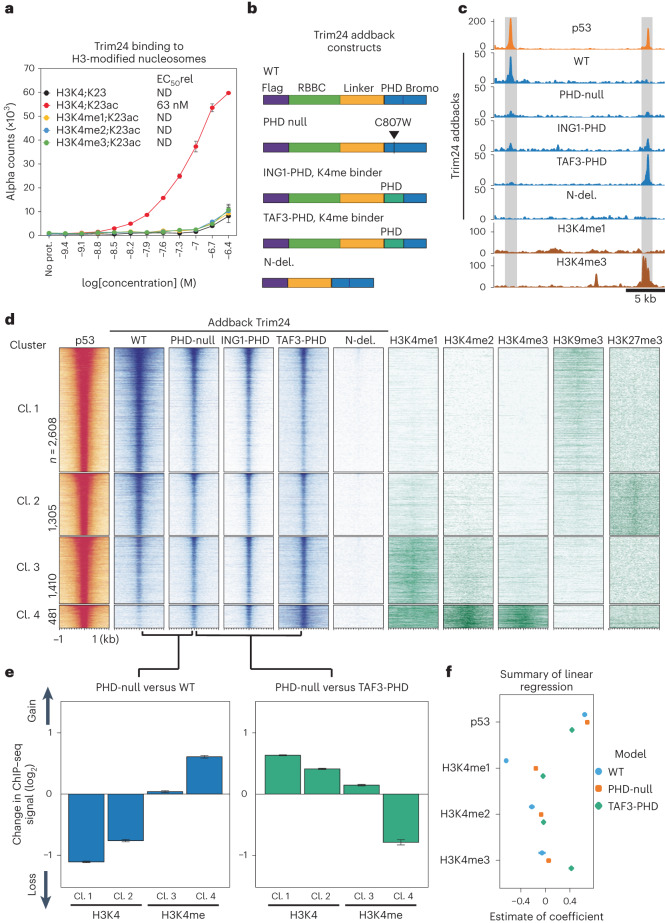
Trim24 affects p53 in an H3K4-methylation-dependent manner. **a**, Binding of recombinant Trim24 to synthetic modified nucleosomes. Values represent the average of two independent experiments; bars represent s.e.m. **b**, Constitutively expressed addback alleles in a *Trim24*-knockout background, including mutations that either nullify the PHD domain or invert its preferences towards methylated H3K4. **c**, Representative regions (gray) of p53 binding and addback Trim24 binding in open and closed chromatin. **d**, K-means clustering of strong p53-binding sites (*n* = 5,804 sites) by histone modifications. Histone marks representative of each cluster are shown (right), alongside binding of p53 and Trim24 variants (left). **e**, Average change in binding (from duplicate experiments) of the PHD-null Trim24 relative to WT (left) and TAF3-PHD variants, for each of the clusters shown in **d**. Mean fold change across loci is displayed; error bars indicate s.e.m. **f**, Estimate of coefficient contributions to a linear regression model of binding for WT, PHD-null and TAF3-PHD variants, using p53 and H3K4 methylation enrichment as variables. del., deletion; EC_50_, half maximal effective concentration; ND, not determinable; No prot., no protein control.

We tested the interaction specificity of the histone-binding domains of recombinant full-length mouse Trim24 towards a panel of modified nucleosomes via an amplified luminescence proximity homogeneous assay. Consistent with previous reports on the isolated PHD/bromo-domain and histone-tail peptides, we detected high enrichment of H3K23ac-modified nucleosomes, but only when H3K4 was not methylated^30^ (Fig. 3a and Supplementary Fig. 9a). In this in vitro assay, Trim24 only bound when both preferred substrates for the PHD domain (H3K4 unmethylated) and bromo-domain (H3K23 acetylated) were present, suggesting that these marks are required for histone engagement.

However, in the cellular context, Trim24-bound sites showed no enriched signal for H3K23ac or any other active histone mark (Supplementary Fig. 9b,c). This was different from the case of unmethylated H3K4, which was indeed highly prominent at Trim24-bound sites (Supplementary Fig. 9b,c), and indicates the possibility that the PHD domain of Trim24 could contribute to its genomic binding at p53 sites. We tested this hypothesis with a set of Trim24 mutants, which we ectopically expressed in a *Trim24*-knockout background. Tested variants included a PHD-null point mutant^30^ and PHD-domain substitutions from ING1 and TAF3 proteins that bind only methylated H3K4 (refs. ^32–34^) (Fig. 3b,c and Supplementary Fig. 10a), as well as the WT Trim24 as a control. In this complementation assay, WT Trim24 showed genomic binding comparable with that of endogenous Trim24, albeit with a slightly reduced signal, suggesting that p53 and chromatin binding preferences were recapitulated (Supplementary Fig. 10b). The N-terminal deletion variant did not bind p53 sites, suggesting that this part of Trim24 is the p53-interaction point.

Next, we asked how the PHD-domain variants influence Trim24 binding as a function of histone modifications. A single point mutation in the PHD domain that blocked the H3K4 interaction resulted in decreased binding to closed chromatin and increased binding to open chromatin, both at the single locus level and globally at p53 sites (Fig. 3c–f and Supplementary Fig. 10c). Although this indicates that the PHD domain is required for chromatin sensitivity, it further suggests that the specificity of the PHD domain is necessary for modulating binding at p53 sites. To test this, we examined binding of Trim24 with heterologous PHD domains possessing different specificities: that of ING1, reported to bind to HK4me2 and HK4me3, and that of TAF3, reported to preferentially bind H3K4me3 (refs. ^32–34^). Indeed, when we embedded these two domains in the Trim24 protein, they were sufficient to redirect Trim24. Importantly, this happened in a chromatin-sensitive manner, leading to increased binding to regions of H3K4 methylation and thus inverting the preference of Trim24 for unmethylated H3K4 (Fig. 3d–f). In summary, the PHD domain is necessary and sufficient for the observed chromatin sensitivity of Trim24.

Next we asked whether Trim24 binding and the impact on p53 might be modulated by other chromatin marks characteristic of heterochromatin. More specifically, we focused on H3K9me3, which is involved in repeat silencing, and H3K27me3, a critical component of repression by the Polycomb system^35,36^. We found that p53 could bind to regions with either of these features, at least in mES cells (Supplementary Fig. 11a–d). Profiling ATAC–seq signals upon activation demonstrated that sites enriched in these marks respond to p53 activation and Trim24 degradation with increased accessibility (Supplementary Fig. 11e,f). Together, these data suggest that H3K4 methylation readout by the PHD domain fully accounts for Trim24 sensitivity to open chromatin by increasing its affinity to closed chromatin sites, where it then modulates p53 function.

### Trim24 affects transcriptional response to p53 activation

**Fig. 4:**
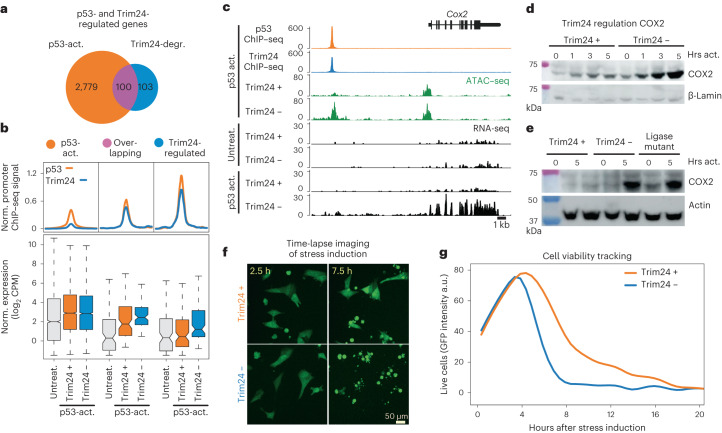
Trim24 regulates a subset of p53 target genes. **a**, Number and overlap of genes that change upon p53 activation compared with uninduced cells and upon Trim24 degradation in p53-active conditions. **b**, p53 and Trim24 ChIP–seq signals (top) in promoter peaks of differentially expressed genes as in **a**, alongside gene expression (bottom) in uninduced and p53-activated conditions with and without Trim24. Averages from triplicate experiments are shown. Center median to first and third quartile, whiskers to 1.5 multiplied by interquartile range. **c**, Representative Trim24-regulated gene *Cox2*. p53 and Trim24 binding and accessibility are shown, with RNA-seq tracks under either uninduced or p53-activated conditions and with or without Trim24 loss. **d**, Western blot of COX2 protein expression after p53 activation (hours after activation (Hrs act.)), with and without Trim24. **e**, Western blot of COX2 protein expression after p53 activation, with and without Trim24 and in the ligase-null mutant (C52/55A) Trim24 variant line. **f**,**g**, Live imaging of GFP-expressing mES cells, measuring cell viability across a 20-h period following stress induction with or without Trim24 degradation. Representative images at 2.5 and 7.5 h after stress induction are shown (**f**), and cell viability as a function of Trim24, quantified for 20 h following stress induction (**g**). Source data

As Trim24 modulates p53-dependent accessibility, we asked whether it contributes to p53 target gene regulation in the Trim24 dTag line. We profiled expression upon p53 activation in the absence or presence of Trim24 (Fig. 4a and Supplementary Fig. 12a). Trim24 loss resulted in 203 genes responding differently to p53 activation (Supplementary Fig. 12b–g). These were bound by p53 and Trim24 at promoter sites (Fig. 4b). Half of these genes were identified as p53-responding genes upon stress induction in the presence of Trim24 and showed an enhanced response in the absence of Trim24. The other half showed only a very weak p53 response that was specifically enhanced in the absence of Trim24. We conclude that Trim24 responsive genes at the transcriptional level are a defined subset of p53 target genes, in line with our observation of Trim24 binding and changes in accessibility. Importantly, Trim24 targets are transcriptionally inactive in p53-uninduced conditions and tend to respond weakly or not at all to activation, supporting a model whereby Trim24 represses p53 targets in closed chromatin.

**Extended Data Fig. 2:**
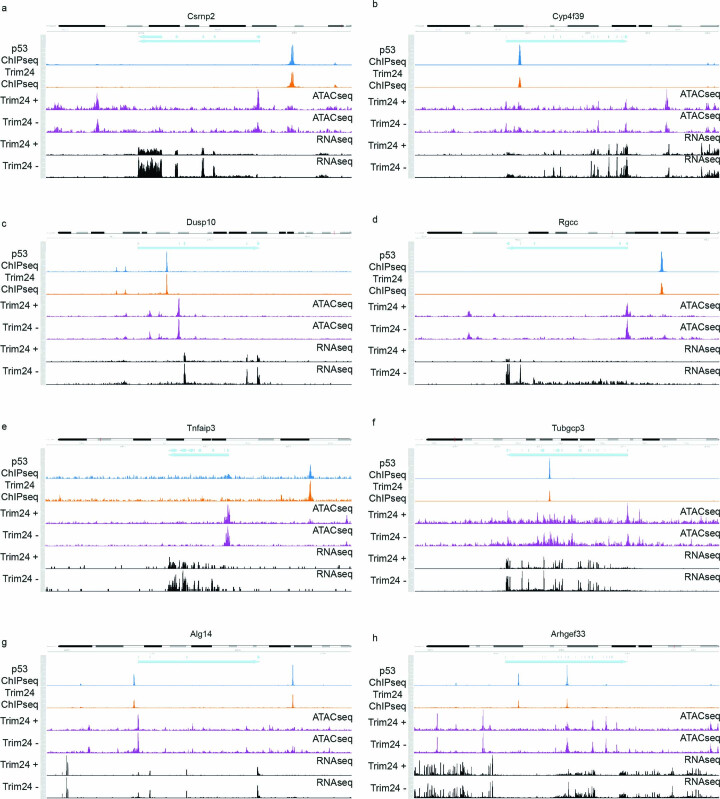
Trim24-regulated genes. (**a**–**h**) Representative Trim24 regulated genes. Shown is p53 and Trim24 binding (top), accessibility (ATACseq, middle) and RNAseq (black, bottom) upon cell stress and with or without Trim24 degradation. Gene location (long arrow) and exons (short arrows) are as indicated for each gene as labelled.

Given that Trim24 has previously been linked to the repression of repetitive DNA elements, we profiled annotated mouse repeats and identified six subtypes that increased in expression following removal of Trim24 (ref. ^31^) (Supplementary Fig. 12h). This included the RLTR1B, RLTR1F and MMGLN elements, which possess p53-binding sites in their LTR promoter sequences (Supplementary Fig. 12i). We note that these only represent a minority of repeats previously reported to be repressed by p53 (refs. ^37,38^). Trim24-repressed targets included several genes involved in inflammatory response and cell cycle regulation, including cyclooxygenase 2 (*Cox2*), regulator of cell cycle (*Rgcc*) and tumor necrosis factor alpha-induced protein 3 (*TNFAIP3*) (Supplementary Fig. 12f and Extended Data Fig. 2). *Cox2* is a key therapeutic target of antiinflammatory drugs and is known to be silent unless induced by inflammatory stimuli^39^ (Fig. 4c,d). The response of Cox2 to p53 activation in stem cells is strongly repressed by Trim24, as in its absence there is at least a nine-fold increase in expression both at the RNA and protein level.

### The RING domain is required for Trim24 function

The N-terminal RING domain is a characteristic feature of TRIM proteins and in many cases confers E3 ubiquitin ligase activity^40^. However, it has been difficult to identify substrates and to separate the enzymatic activity of TRIM proteins from their potential scaffolding function; this is most evident for the highly studied Trim28, which recruits Setdb1 to catalyze repressive H3K9me3 for silencing^41^. To determine the role of the RING domain in Trim24, we generated a specific point mutation at the endogenous gene using CRISPR–Cas9. This should result in a ubiquitin-ligase-null mutant version of the protein, as previously shown for Trim proteins^42,43^. Next, we studied the impact of this RING-domain mutant on p53-dependent gene activation and found that it failed to efficiently silence Trim24-repressed genes, suggesting its requirement for Trim24 function (Fig. 4e and Supplementary Fig. 13a–d). Importantly, a ChIP–seq comparison revealed highly similar binding of WT and C52/55A-mutant Trim24 at p53 sites (Supplementary Fig. 13e,f).

### Trim24 contributes to cell viability upon stress

We used live-cell imaging over a 40-h time course of p53 activation with loss of Trim24 to examine whether Trim24 target genes contribute to the phenotypic stress response in mES cells (Supplementary Fig. 14a–h). Under stress conditions, the absence of Trim24 resulted in a rapid decrease in numbers of live cells, whereas Trim24-expressing cells persisted for twice as long before reaching similar levels (Fig. 4f,g, Supplementary Fig. 14h,i and Supplementary Video 1). Under p53-uninduced conditions, loss of Trim24 slightly decreased proliferation (Supplementary Fig. 14g and Supplementary Video 2). Furthermore, genetic deletion of *p53* in the Trim24 degron line demonstrated that the effect on viability after Trim24 loss requires p53 to be present in cells (Supplementary Fig. 14j).

### Trim24 functions in neurons

To determine whether Trim24-mediated repression of p53 is conserved in a distinct and postmitotic cellular context we used a neuronal differentiation system in the Trim24 degron background^28,44^. This revealed that binding of Trim24 was similarly highly correlated with p53 in closed chromatin sites, confirming the observed interactions in stem cells (Supplementary Fig. 15a). The p53 response to stress was overall lower in neurons, probably reflecting reduced p53 levels (Supplementary Fig. 15b–d). The reduced p53 activation also dampened the Trim24 effect, which was present but to a lesser degree (Supplementary Fig. 15e,f). Indeed, binding of p53 and Trim24 scaled well between cell types but was generally less enriched in neurons (Supplementary Fig. 15g). Together, these data suggest that Trim24 generally limits the activation potential of a subset of initially silent p53 target genes and that co-occupancy is conserved in stem cells and postmitotic neurons, where it limits p53 activity in closed chromatin.

## Discussion

Here, we demonstrate that Trim24 bridges p53 activity to the local chromatin state via its interaction with p53 and unmethylated H3K4. p53 has the unusual characteristic of binding its motifs embedded in heterochromatin, yet its ability to open chromatin and activate genes upon stimulation is affected by local histone modifications in a Trim24-dependent process. In this context, it is important to note that almost no TFs possess known protein domains that recognize histone modifications, such as PHD domains, bromo-domains, chromo-domains, etc., suggesting that TFs themselves generally do not directly interact with chromatin marks^3^. Our work provides an example of how a specific cofactor, Trim24, connects a TF with histone modifications. The activity of p53 and its target genes are tightly controlled, and mediating such context-dependent activation might be particularly relevant for TFs that can engage heterochromatin. In the case of Trim24, this could serve as a mechanism to locally threshold p53 response or to enable cell-type-specific responses that vary with changes in K4 methylation, which are well described for cell-type-specific enhancers, for example^45^.

Trim24 has previously been linked to cancer, showing elevated expression in several types including breast cancer, whereas gain of expression in mice leads to elevated rates of cancer^30,46–48^. This has motivated the development of Trim24-degrader compounds^49^. It is tempting to speculate that Trim24 might have an effect on specific p53-target genes in cancer, given the findings here demonstrating the mutual contribution of H3K4 methylation and p53 to gene expression.

We identify *Cox2* as a prominent Trim24-regulated gene (Fig. 4c). It encodes a highly clinically relevant enzyme that catalyzes the first, rate-limiting step of prostanoid formation and is the target for nonsteroidal antiinflammatory COX2 inhibitors, indicating roles in pain, fever, inflammation and tumorigenesis^39^. A key theme in *Cox2* function is its role as an inducible gene activated upon inflammatory stimuli, in keeping with the function of Trim24 in repressing p53 response genes that are initially inactive (Fig. 4b). This suggests the need to tightly modulate *Cox2* activation; indeed, overexpression of *Cox2* or prolonged use of COX2-selective inhibitors results in carcinogenesis and cardiotoxicity^50^. Our results suggest that the effects of Trim24 demonstrated here would scale with the potential for p53 activation, which varies between tissues. Trim24 is expressed highly in the ovary and has been shown to promote proliferation in a mouse epithelial ovarian model of tumorigenicity, a tissue that is notably high in p53 levels and p53-response^51–53^. As loss of Trim24 decreases cell viability upon stress, this indicates the possibility that regulation of the dosage of p53 targets such as *Cox2* is required for a normal stress response.

Although p53 was responsible for the majority of Trim24-binding sites in mES cells, there are several reports of additional Trim24-interacting TFs in various cellular contexts^48,54^. Whether these additional interactions are sensitive to closed chromatin remains to be determined, although the potential for this is likely to be higher during cellular transitions such as differentiation, when newly active TFs initially encounter closed chromatin. Trim24 has been reported to have a role in the silencing of repetitive elements in hepatocytes^31^, similar to its paralogs Trim28 and Trim33, which are recruited by TFs to silence specific repetitive elements^41,55^. Here, we observed a specific set of repeats with strong p53 motifs that become activated in mouse stem cells after Trim24 loss, arguing for a highly targeted repression system that is TF-dependent and context-dependent. Trim24 is present at target loci but seems to only function upon p53 activation.

Trim24 loss did not alter p53 occupancy in closed chromatin, and the nature of stable p53 binding at these regions remains to be determined (Fig. 2b). Although other studies have reported that repressive chromatin features such as H3K9me3 are inherently restrictive for TF binding^56^, several high-affinity p53-binding sites co-occupy these marks, and these are made accessible by p53 activation (Supplementary Fig. 11a–f). Our results suggest that in the case of p53, specific chromatin marks can limit the ability of a TF to open chromatin, thereby restricting TF functions in a manner distinct from simply blocking DNA binding.

We note the discrepancy between Trim24-nucleosome binding in vitro, where the bromo-domain H3K23ac shows synergistic binding with the PHD domain H3K4, and binding in vivo where H3K23ac appears to be absent from Trim24-binding sites (Supplementary Fig. 9a–c). It seems possible that p53–DNA interactions provide the necessary stability for the PHD domain to individually contribute, although it remains to be determined how this seemingly breaks the synergism between PHD domain and bromo-domain.

It also remains to be determined how Trim24 exerts a repressive function on p53, as it does not appear to affect local p53 levels. Trim24 has previously been implicated as a ubiquitin ligase of p53 (ref. ^29^), and its RING domain is required in human cancer cell line models^49^. It has proven challenging to characterize E3 ligase substrates at endogenous protein levels^57^ and to separate ligase activity from scaffolding function as in the case of Trim28 (ref. ^58^). Here, we show that Trim24 requires a WT RING domain to regulate p53 (Fig. 4e and Supplementary Fig. 13a–d). Trim24 has recently been demonstrated to enable K63-linked ubiquitination; while K48-linked ubiquitination is associated with degradation, K63-linked ubiquitination is instead associated with modulation of protein–protein interactions^59,60^. We were able to recapitulate Trim24-dependent effects upon activation of p53 via inhibition of Mdm2, which catalyzes K48-linked ubiquitination (Supplementary Fig. 7g), indicating that Mdm2-dependent and Trim24-dependent ligase activities on p53 are functionally distinct. It remains to be determined how Trim24 ligase activity modulates p53 activity or how such posttranslation modifications influence p53 interactions with any of the several coactivator proteins previously described^61,62^.

Our findings reveal a mechanism by which TF regulation is accomplished locally on chromatin, representing a convincing instance where a histone mark is functionally linked to TF potency by the actions of a single histone-interaction domain. It seems likely that other chromatin-interacting proteins with histone-interacting domains could have comparable roles; this should be testable using a combination of high-resolution readouts and precise functional models such as those we have applied here.

## Methods

### Data reporting

No statistical methods were used to predetermine sample size. The experiments were not randomized and the investigators were not blinded to allocation during experiments or outcome assessment.

### Cell culture

#### Mouse ES cells

Mouse ES cells (TC-1 line, background 129S6/SvEvTac, originally obtained from A. Dean at the National Institutes of Health), with a recombinase-mediated cassette exchange (RMCE) site located in the gamma-globin locus, were used as WT cells^63^. These were used for the generation of p53 and Trim24 degron lines, as well as Ngn2-induction, Trim24-addback and GFP-expressing lines. The DNMT triple-knockout cell line was generated from this line. Mouse ES cells were cultured as described previously^64^. Briefly, cells were maintained in Dulbecco’s modified Eagle medium (DMEM, Invitrogen), supplemented with 15% fetal calf serum (Invitrogen), l-glutamine (Gibco) and nonessential amino acids (Gibco), betamercaptoethanol (Sigma) and leukemia inhibitory factor (produced in-house). Experiments were performed with cells grown for several passages on plates coated with 0.2% gelatin (Sigma).

#### Human cells

The human primary cell line (HMEC) was cultured in mammary epithelial cell growth medium (MEGM, Gibco) supplemented with growth factors (MEGM-SingleQuots CC-4136, Gibco) bovine pituitary extract (BPE), hydrocortisone, human epidermal growth factor (hEGF), insulin and gentamicin/amphotericin-B (37 °C, 7% CO_2_). These cells (CC-2551) were obtained from Lonza.

#### Insect cells

Sf9 cells were purchased from Thermo Fisher Scientific (catalog no.: 11496-015).

#### Generation of cell lines

We tagged the endogenous *p53* and *Trim24* genes with the V5 epitope tag to facilitate detection and with the FKBP12(F36V) variant to induce degradation upon addition of the dTAG13 compound^26^. A V5-FKBP12(F36V) gene fragment was ordered with 100 bp homology arms to the N terminus of the mouse *p53* gene, and an FKBP12(F36V)-V5 gene fragment was ordered with 100 bp homology arms to the carboxyl terminus of the mouse *Trim24* gene (Twist Biosciences). A guide RNA (gRNA) sequence taken from the *Escherichia coli* genome (GTGTTGTGGACTGCGGCGGTCGG) and restriction enzyme sites were added to either end of the gene fragment via PCR. The PCR product was gel purified using a QIAquick Gel Extraction Kit (Qiagen), digested and cloned into a donor vector. gRNA oligos against the N terminus of the *p53* gene (ATCCGACTGTGACTCCTCCA) and the C terminus of the *Trim24* gene (GGCGGCGTTACTTAAGCAGC) and the bacterial gRNA were ordered (Microsynth); these were annealed and cloned into the BsaI sites of the pC2P plasmid, which contains a Cas9–P2A–puromycin cassette. WT cells (5 × 10^5^) were cotransfected in suspension using Lipofectamine 3000 (Thermo Fisher Scientific) with 750 ng of the donor plasmid, 250 ng of the target gRNA plasmid and 100 ng of the bacterial gRNA plasmid. They were plated in a six-well plate coated with 0.2% gelatin and left for 24 h. After this time, the medium was replaced with medium containing puromycin (2 nM), and the cells were left for another 24 h, after which the puromycin-containing medium was replaced with normal medium, and cells were left to recover for 24–48 h in the normal medium. Cells were then plated for clone picking and left to grow for approximately 7 days. Individual clones were picked into a 96-well plate and genotyped by PCR. Clones that showed a homozygous knock-in at the genetic level were expanded and verified by western blotting for integration of V5 and FKBP12(F36V) before and after degradation. In addition, from the same pool of cells, a subset of clones was found to have lost expression of the targeted gene owing to Crispr–Cas9 cleavage without repairing-in the tagged sequence, resulting in mutations that caused a null allele of the endogenous gene. These knockout alleles were expanded and verified by western blotting.

We edited the endogenous *Trim24* gene (NP 001258993.1) to harbor CA mutations C52A and C55A in the RING domain of Trim24, rendering homologous Trim proteins incompatible with ubiquitin ligase activity^42^. A C52/55A double mutation gene fragment was ordered with 100 bp homology arms to the RING domain of the mouse *Trim24* gene (Twist Biosciences). Gene editing was performed as for the introduction of V5 and FKBP12(F36V) sequences, using a gRNA oligo against the RING domain of *Trim24* (ACACGGCGCAAGTGTCCAAC).

To generate addback alleles of the *Trim24* gene, RMCE was used to generate pools of cells expressing gene fragments downstream of a Cag promoter, inserted at the same genomic locus^63,65^. Geneblocks (Twist Biosciences) were ordered and cloned into a donor plasmid, including full-length Trim24 (NP 001258993.1), an N-terminal fragment containing the RBBC domain (amino acids 1–473), an N-terminal deletion (amino acids 393–1045), a PHD-null mutation variant (C807W), an RING-domain mutant variant (C52A and C55A), and variants where the PHD domain (amino acids 793–838) was replaced with that of ING1 (amino acids 141–190, NP_937861.1) or TAF3 (amino acids 856–901, NP_114129.1). In brief, *Trim24*-knockout TC-1 ES cells (background 129S6/SvEvTac) carrying an RMCE selection cassette (described previously^63^) were selected under hygromycin (250 μg ml^−1^, Roche) for 10 days. Next, 4 million cells were electroporated (Amaxa Nucleofection, Lonza) with 25 μg L1-Cag/FLAG_NLS_ORF-1L plasmid and 15 μg pIC-Cre. Negative selection with 3 μM ganciclovir (Roche) was started 2 days after transfection and continued for 10 days. Individual clones were picked into a 96-well plate and genotyped by PCR. Clones that showed integration at the genetic level were expanded and verified by western blotting. To generate GFP-expressing cells, RMCE was used on the V5dTag-Trim24 mES cell line to generate pools of cells expressing GFP downstream of a Cag promoter, inserted at the same genomic locus^63,65^. RMCE and clone selection were performed as described above.

To induce the rapid differentiation of ES cells into neurons, a PiggyBac construct carrying an inducible *Ngn2* gene (CAG:rtTA,TetO:Ngn2-T2A-GFP) was randomly integrated into the genome^44^. *Trim24* degron mES cells were nucleofected with the PiggyBac construct and a dual helper plasmid (expressing transposase). After 48 h, cells were selected with G418 (300 μg ml^−1^) for 7 days before plating for clone selection. Clones were picked and screened for their ability to rapidly differentiate into neurons as previously described^28^.

#### Neuronal differentiation

Neurons were generated by differentiating mES cells as previously described^28^. Briefly, dishes were coated with sterile filtered (0.22 μm) poly-d-lysine hydrobromide (0.5 mg ml^−1^, Sigma, P-7886-100MG) diluted in borate buffer (50 mM, pH 8.5) overnight at 37 °C, washed three times with sterile water and coated for at least 2 h with laminin (6.65 μg ml^−1^, Sigma, L2020-1MG) in phosphate-buffered saline (PBS) overnight at 37 °C. ES cells were cultured as above, trypsinated and plated at a density of 2 × 10^6^ per 10-cm dish in proliferation medium (DMEM/F12 with Glutamax (LifeTech 31331-028), 1× B27 supplement without vitamin A (LifeTech 12587-010), 1× N2 supplement (LifeTech 17502-048)) supplemented with 1 μg ml^−1^ doxycycline.

#### ChIP–seq

Mouse ES cells (1.5l × 10^7^) were seeded into 15-cm plates the day before the experiment. Neurons were differentiated by seeding mES cells (0.75 × 10^7^) into 15-cm plates 3 days before the experiment in proliferation media supplemented with 1 μg ml^−1^ doxycycline. Where applicable, the media were exchanged with fresh media containing 1 µM doxorubicin (44583, Sigma-Aldrich) and/or dTAG13 compound (500 nM, Tocris) in the morning 4 h before harvesting of cells. ChIP was carried out as previously described^66^ with the following modifications: (1) chromatin was sonicated for 22 cycles of 20 s on and 40 s off using a Diagenode Bioruptor Pico; (2) 75 μg of chromatin was used per immunoprecipitation; (3) protein A magnetic Dynabeads (Thermo Fisher Scientific) were used. Immunoprecipitated DNA was subjected to library preparation (NEBNext Ultra II DNA Library Prep Kit, Illumina). In the library preparation protocol, samples were amplified using 12 PCR cycles. Libraries were sequenced on an Illumina HiSeq (50 cycles) or NextSeq (paired-end, 75 cycles). For control datasets, anti-IgG (M7023, Sigma-Aldrich) was used to control for bead and antibody-unspecific enrichments.

#### RNA sequencing

Mouse ES cells were seeded into six-well plates (2.5 × 10^5^ cells per well) the day before the experiment. Where applicable, the media were exchanged with fresh media containing 1 µM doxorubicin (44583, Sigma-Aldrich) and/or dTAG13 compound (500 nM, Tocris) for the indicated amount of time (2, 4, 8 or 12 h) before harvesting. RNA was isolated using a Direct-zol MicroPrep RNA Purification Kit (Zymo), following the manufacturer’s instructions. Sequencing libraries were prepared from 100 ng of purified total RNA for biological replicates using TruSeq stranded Total RNA Library Prep (Illumina). Libraries were sequenced on an Illumina HiSeq (50 cycles) or NovaSeq (paired-end, 100 cycles).

#### ATAC–seq

ATAC–seq was performed according to the previously described protocol^64,67^ with modifications. Mouse ES cells were seeded into six-well plates (2.5 × 10^5^ cells per well) the day before the experiment. Neurons were differentiated by seeding mES cells (0.2 × 10^6^) into six-well plates three days before the experiment in proliferation medium supplemented with 1 μg ml^−1^ doxycycline. Where applicable, the media were exchanged with fresh media containing 1 µM doxorubicin (44583, Sigma-Aldrich) to activate p53, and/or dTAG13 compound (500 nM, Tocris) to degrade dTAG-tagged proteins, for the indicated amount of time (1, 2, 4, 8 or 12 h) before harvesting. For activation of p53 via alternative pathways, media were exchanged with fresh media containing 20 µM nutlin3a (444152, Sigma-Aldrich) for 4 h, or cells in culture media were subjected to 60 Gy ionizing radiation using an automated CellRad system (Faxitron), and left for 4 h. In brief, 5 × 10^4^ cells were resuspended in 1 ml of cold ATAC–seq resuspension buffer (RSB: 10 mM Tris-Cl pH 7.4, 10 mM NaCl and 3 mM MgCl_2_ in water). Cells were centrifuged at 500*g* for 5 min in a prechilled centrifuge (4 °C). Cell pellets were suspended in 50 μl ATAC–seq RSB containing 0.1% NP40, 0.1% Tween-20 and 0.01% digitonin by pipetting up and down three times. This cell lysis reaction was incubated on ice for 3 min. After lysis, 1 ml of ATAC–seq RSB containing 0.1% Tween-20 (without NP40 or digitonin) was added, and the tubes were inverted for mixing. Nuclei were then centrifuged for 10 min at 500*g* (4 °C). Nuclei were suspended in 50 μl Tn5 transposition mix (10 μl 5× reaction buffer (50 mM Tris-Cl pH 8.5, 25 mM MgCl_2_, 50% dimethylformamide), 2.5 μl transposase (100 nM final), 16.5 µl PBS, 0.5 µl 1% digitonin, 0.5 µl 10% Tween-20, 20 µl water) by pipetting up and down six times. Transposition reactions were incubated at 37 °C for 30 min in a thermomixer with shaking at 1,000 rpm. Reactions were cleaned up using a MinElute PCR Purification Kit (Qiagen). The eluted transposed DNA was subjected to PCR amplification using Q5 High-Fidelity Polymerase (NEB) for seven cycles. Libraries were sequenced on an Illumina NextSeq (paired-end, 75 cycles).

#### Immunofluorescence microscopy

Mouse ES cells were seeded on poly-l-lysine-coated eight-well (8 × 10^4^ cells per well) μ-Slides (Ibidi) and left for 4 h before the medium was exchanged for fresh medium containing 1 µM doxorubicin (44583, Sigma-Aldrich) or 20 μM nutlin3a (444152, Sigma-Aldrich) and/or dTAG13 compound (500 nM, Tocris) and left for another 4 h to activate p53 and/or degrade Trim24. Cells were fixed with 4% formaldehyde in PBS for 15 min, washed three times with PBS, permeabilized in PBS with 5% fetal calf serum (Invitrogen) and 0.3% Triton X-100 for 30 min, then incubated overnight with antibodies (Supplementary Table 1) in PBS with 1% bovine serum albumin (BSA) and 0.1% Tween-20. On the next day, plates were washed three times with PBS with 1% BSA and 0.1% Tween-20 and incubated with secondary antibodies (1:500) in PBS with 1% BSA and 0.1% Tween-20 for 1 h at room temperature. Plates were incubated for 10 min with PBS with Hoechst 33342 (1:500 dilution) (134406, Thermo Fisher Scientific) and then washed three times with PBS. Cells were imaged with a Visitron Spinning Disk W1 microscope with a ×40 objective. Images were processed with ImageJ: the background was subtracted, nuclei were segmented and the mean intensity was measured.

#### Time-lapse microscopy

Mouse ES cells were seeded on poly-l-lysine-coated eight-well (3 × 10^4^ cells per well) μ-Slides (Ibidi) and left for 4 h before the medium was exchanged for fresh medium containing 1 µM doxorubicin (44583, Sigma-Aldrich) and/or dTAG13 compound (500 nM, Tocris). Cells were kept at 37 °C and 7% CO_2_ throughout the experiment. Cytoplasmic GFP-expressing cells were live-imaged with a Visitron Spinning Disk W1 microscope using a 488-nm laser and ×20 objective in 12-min intervals. At least six imaging areas were recorded per condition in two different wells. Images were processed with ImageJ. Background-subtracted images were segmented, separating (round, detached, bright) dead cells from the (plate-attached, flat) live cells. The intensities from each of the imaging areas over time were plotted using the R package ggplot2 (ref. ^68^) (line plot from loess fit with 0.95 confidence interval for all replicates). The quantification method for the time-lapse cell viability assay was confirmed by quantifying the number of cells in Hoechst 33342- and propidium iodide-stained (Thermo Fisher I34406, V13242) single timepoint images with different cell densities and treatments, where the measured total GFP signal of the attached/live cells per imaging area (as in the time-lapse analysis) correlates with the attached/live cell number (Supplementary Fig. 14a–e).

#### Coimmunoprecipitation and mass spectrometry

Mouse ES cells were seeded into 15-cm plates (1.5 × 10^7^ cells) the day before the experiment. Where applicable, the medium was exchanged for fresh medium containing 1 µM doxorubicin (44583, Sigma-Aldrich) and/or dTAG13 compound (500 nM, Tocris) for 4 h before harvesting. In brief, cells were washed with PBS and harvested by treatment with 0.05% trypsin. Cells were pelleted by centrifuging for 2 min at 300*g* and resuspended in 3 ml PBS with 0.1% formaldehyde. After 10 min of cross-linking, reactions were quenched with 150 µl of 2.5 M glycine, vortexed briefly and left on ice for 2 min. Cells were pelleted by centrifuging for 3 min at 4 °C at 500*g* and washed in 1.5 ml PBS with 0.2% BSA, before being repelleted by centrifuging for 3 min at 4 °C at 500*g* and resuspended in 180 µl TMS buffer (10 mM Tris-Cl pH 8, 1 mM MgCl_2_, 1% sodium dodecyl sulfate (SDS), 1× Complete Protease Inhibitor Cocktail (Roche)) with 0.4 µl benzonase (E1014, Millipore) prechilled at 12 °C. Samples were incubated at 12 °C for 30 min in a thermomixer with shaking at 500 rpm; then 1,570 µl ice-cold dilution buffer (11.4 mM Tris-Cl pH 8, 573 mM NaCl, 1.14% Triton X-100, 11.46 mM EDTA, 1× Complete Protease Inhibitor Cocktail (Roche)) was added, and the samples were left on ice for 5 min. Samples were centrifuged at 16,000*g* at 4 °C for 5 min and the supernatants were transferred to new tubes with 10 µl anti-V5 monoclonal antibody magnetic beads (M215-11, MBL). V5-tagged proteins were bound to beads by incubation for 2 h in an overhead rotator and washed three times with 1 ml ice-cold wash buffer (10 mM Tris-Cl pH 8, 500 mM NaCl, 1% Triton X-100, 0.1% SDS, 1 mM EDTA). Beads were resuspended in 5 µl digestion buffer (3 M guanidine-HCl, 20 mM EPPS pH 8.5, 10 mM CAA, 5 mM TCEP) + 1 µl Lys-C and incubated at room temperature for 4 h. Beads were mixed with 17 µl 50 mM HEPES pH 8.5; then, 1 µl 0.2 µg µl^−1^ trypsin was added, followed by incubation overnight at 37 °C. The following morning, another 1 µl of 0.2 µg µl^−1^ trypsin was added, and the digestion was continued for an additional 5 h. Samples were acidified by addition of 1 µl of 20% trifluoroacetic acid and sonicated in an ultrasound bath. Peptides were analyzed by liquid chromatography with tandem mass spectrometry on an EASY-nLC 1000 (Thermo Scientific) with a two-column set-up. The peptides were applied onto a peptide μPAC trapping column in 0.1% formic acid, 2% acetonitrile in H_2_O at a constant flow rate of 5 μl min^−1^. Using a flow rate of 500 nl min^−1^, peptides were separated at room temperature with a linear gradient of 3–6% buffer B in buffer A in 4 min, followed by a linear increase from 6 to 22% in 55 min, 22–40% in 4 min, and 40–80% in 1 min. The column was finally washed for 13 min with 80% buffer B in buffer A (buffer A: 0.1% formic acid; buffer B: 0.1% formic acid in acetonitrile) on a 50-cm μPAC column (PharmaFluidics) mounted on an EASY-Spray source (Thermo Scientific) connected to an Orbitrap Fusion LUMOS (Thermo Scientific). The data were acquired using 120,000 resolution for the peptide measurements in the Orbitrap and a top T (3 s) method with HCD fragmentation for each precursor and fragment measurement in the ion trap according to the recommendation of the manufacturer (Thermo Scientific).

#### Recombinant Trim24 and p53 protein expression

Mouse full-length TRIM24 and truncations (C-terminal domain: amino acids 813–1051; N-terminal domain: amino acids 1–473; bonsai: deletion of amino acids 474–757) were subcloned into a pAC-derived vector^69^ containing an N-terminal Flag-tag. Mouse full-length p53 was subcloned into a pAC-derived vector^69^ containing an N-terminal Strep-tag. Recombinant protein was expressed in *Spodoptera frugiperda* SF9 cells using the Bac-to-Bac system (Thermo Fisher). Cells were cultured at 27 °C and harvested 2 days after infection.

Cells expressing Trim24 were resuspended in lysis buffer (50 mM HEPES pH 8.0, 225 mM NaCl, 0.1% Triton X-100, 1× protease inhibitor cocktail (Sigma)) and lysed by sonication. The supernatant was harvested by ultracentrifugation (290,000*g*, 45 min, 4 °C) and the protein was purified by Flag-affinity purification followed by MonoQ anion exchange chromatography (GE Healthcare). Finally, TRIM24 was subjected to size-exclusion chromatography (Superose 6; GE Healthcare) in SEC buffer (20 mM HEPES pH 8.0, 225 mM NaCl, 0.5 mM TCEP, 10% glycerol). The purified protein was concentrated and stored at −80 °C.

Cells expressing p53 were resuspended in lysis buffer (20 mM Tris-HCl pH 8.0, 1 M NaCl, 5% glycerol, 0.5 mM TCEP) and lysed by sonication. The supernatant was harvested after ultracentrifugation (290,000*g*, 45 min, 4 °C) and the protein was purified by Strep affinity purification (Strep-Tactin Sepharose, IBA) followed by heparin ion-exchange chromatography (GE Healthcare). Finally, p53 protein was subjected to size-exclusion chromatography (Superose 6; GE Healthcare) in SEC buffer (20 mM HEPES pH 7.4, 150 mM NaCl, 5% glycerol, 0.5 mM TCEP). The purified protein was concentrated and stored at −80 °C.

#### Pull-down of Trim24 truncations and p53 using purified proteins

Anti-Flag affinity beads (Anti-FLAG M2 Affinity Gel, Sigma) and Strep-Tactin beads (MagStrep ‘type3’ XT beads, IBA) were washed in wash buffer (50 mM HEPES pH 8.0, 150 mM NaCl, 1× protease inhibitor cocktail (Sigma)). Flag-tagged TRIM24 N-terminal and C-terminal domains (0.25 nmol) were mixed with Strep-tagged p53 full-length protein in equimolar ratios and incubated with anti-Flag or Strep-Tactin beads for 2 h at 4 °C. Subsequent to intensive washing using wash buffer, bound proteins were eluted from Flag resin using elution buffer (50 mM HEPES pH 8.0, 150 mM NaCl, 0.5 mg ml^−1^ 3xFlag peptide). Flag elutions and Strep-Tactin beads were boiled in SDS sample buffer and loaded onto a 4–20% TGX gel (Bio-Rad) followed by staining using QuickBlue Protein stain (LubioScience).

#### Coinfection and copurification of Trim24 constructs and p53

Recombinant proteins were expressed in a 10 ml culture of *S. frugiperda* SF9 cells using the Bac-to-Bac system (Thermo Fisher). Cells were cultured at 27 °C, harvested 2 days after infection, resuspended in lysis buffer (50 mM HEPES pH 8.0, 500 mM NaCl, 0.1% Triton X-100, 1× protease inhibitor cocktail (Sigma)) and lysed by sonication. The supernatant was harvested after centrifugation (12,000*g*, 10 min, 4 °C) and split into two vials for affinity purification with anti-Flag beads (Trim24: anti-FLAG M2 Affinity Gel, Sigma) and Strep-Tactin beads (p53: MagStrep ‘type3’ XT beads, IBA). Subsequent to intensive washing using lysis buffer, bound proteins were eluted from Flag resin using elution buffer (50 mM HEPES pH 8.0, 500 mM NaCl, 0.1% Triton X-100, 0.5 mg ml^−1^ 3xFlag peptide). Flag elutions and Strep-Tactin beads were boiled in SDS sample buffer and loaded onto a 4–20% TGX gel (Bio-Rad) followed by staining using QuickBlue Protein stain (LubioScience).

#### Trim24–nucleosome binding assays

To investigate whether specific histone modifications contribute to the ability of Trim24 to engage nucleosomes, we utilized the dCypher approach^70^ via the Alpha platform^71,72^. Semisynthetic nucleosomes and controls with defined posttranslational modifications were synthesized, purified and assembled using the commercial dCypher and versaNuc services (https://www.epicypher.com/services/). Binding assays were performed as previously described^70,73^ with modifications. In brief, a dCypher panel of 74 nucleosomes plus DNA and buffer controls were combined with Flag-Trim24 at 175 nM (the optimal screening concentration as determined by initial binding measurements to candidate nucleosomes) in 10 µl binding buffer (20 mM Tris-Cl pH 7.5, 25 mM NaCl, 0.01% NP40, 0.01% BSA and 1 mM DTT) followed by 30 min incubation at 23 °C in a 384-well plate format. A 10-µl mixture of 1:400 anti-Flag antibody (Millipore Sigma F7425) with 5 μg ml^−1^ Protein A Acceptor beads and 10 μg ml^−1^ Alpha Streptavidin Donor beads was added, followed by incubation at 23 °C in subdued lighting for 60 min. AlphaLISA signal was measured on a PerkinElmer 2104 EnVision (680 nm laser excitation, 570 nm emission filter, ±50 nm bandwidth). To test specific modifications (that is, versaNucs combinatorically modified with H3K4me-0/1/2/3; H3K23ac), binding reactions were carried out and measured in a similar manner, over a Flag-Trim24 dilution series of 0.4 µM to 0.39 nM. All measurements were performed in duplicate, and mean signal values are shown with error bars indicating standard error. Relative half maximal effective concentration values were computed using a four-parameter logistical model in GraphPad Prism 8 as previously described^74,75^.

#### Cell viability assay

Cell viability was assessed using the CellTiter-Glo 2 cell viability assay (Promega) according to the manufacturer’s protocols. Briefly, cells were seeded into 96-well plates (25K per well for mES cells, 10K per well for neurons) and treated with either doxorubicin or dTag for various time periods. Equal volumes of CellTiter-Glo reagent were added to the media, followed by incubation for 2 min in a thermomixer at 500 rpm and then for a further 8 min. Luminescence representing the number of viable cells was measured using a Centro XS^3^ LB 960 (Berthold Technologies) 96-well microplate luminometer, for 0.5 s and with four technical replicate measurements from which average signal values were merged.

### Computational analyses

#### ChIP–seq

ChIP–seq datasets were aligned to either the mm10 mouse or hg19 human assembly using the QuasR^76^ Bioconductor package, which uses Bowtie^77^ (RBowtie package). Alignments were performed with the default settings, allowing for uniquely mapping reads. Peak calling on all datasets was performed with MACS2 (v.2.1.3.3)^78^ using the callpeak argument with default settings and specifying the genome size with -g mm or -hs for mouse or human, respectively. Peaks were called for mouse ChIP–seq datasets using matched IgG ChIP–seq datasets as controls. Peaks were called for human ChIP–seq datasets using either matched IgG ChIP–seq or matched chromatin input sequencing datasets as controls. Peaks from replicate datasets and across different samples were unified by sorting and merging overlapping regions using the bedtools^79^ (v.2.25.0) ‘sort’ and ‘merge’ functions with default settings, as in the case of human p53 ChIP–seq (Supplementary Fig. 2) and Trim24-addback ChIP–seq (Supplementary Fig. 10b,c). Motif enrichments on individual ChIP–seq datasets were performed using the HOMER^80^ software, with each peak set ranked according to MACS2-defined *P* values; the top 500 peaks were used. The HOMER findMotifsGenome.pl function was run on these top peaks, using the -len argument to search for motifs of 10, 12, 14 and 16 bp in length and the -size argument to search for motifs within 250 bp around the peak center. Finding both known and de novo motifs was performed and reported as indicated within figures and visualized using HOMER nucleotide frequencies and the Bioconductor SeqLogo^81^ R package (v.1.64.0). Read counts were generated over defined genomic regions (that is, peak regions) using the QuasR^76^ function qCount after removing blacklisted regions^82^, with default parameters and shifting the reads 80 bp, which was approximately half the size of ChIP–seq library fragments. For datasets with paired-end sequencing, this was instead shifted to half of the fragment length. Briefly, counts were normalized between the datasets being compared, a pseudocount of 8 was added and data were log_2_ transformed. Normalization was performed by multiplying counts by a scaling factor that was determined by the library with the lowest number of mapped reads between the datasets, that is, scaled down to the smaller library: scaling factor χ = min(Sample 1, Sample 2,…, Sample χ)/Sample χ, where Sample 1, Sample 2,…, Sample χ are the total numbers of mapped reads in each respective sample. The pseudocount of 8 was used to decrease noise at low read counts between samples. Enrichments of log_2_ ChIP–seq read counts were calculated by subtracting the matched log_2_ counts from corresponding control datasets. Similarly, changes in binding were calculated based on differences in log_2_ ChIP–seq read counts between datasets. To define a reference set of p53 and Trim24 peaks from the replicate experiments in parental mES cell lines with or without p53 activation, consensus peak calling was carried out as follows: MACS2 peaks from individual replicates were assembled using R package DiffBind^83^ (v.3.2.4) to generate a nonoverlapping set of genomic peaks. As described in the Encode project^84^, irreproducibility discovery rate^85^ (IDR) analysis was used to define a reliable set of consensus peaks using a threshold of IDR < 0.01, average ChIP–seq enrichment >2 and a minimum ChIP–seq enrichment of 0.5 in each replicate. Heatmaps were generated by counting the 5′ positions of mapped reads relative to defined genomic regions (that is, peak regions) using the QuasR^76^ function qProfile and visualized using the EnrichedHeatmap^86^ Bioconductor package (v.1.26.0). In brief, qProfile was run with default parameters for 1-kb regions centered on the middle of each peak region and shifting the reads by 80 bp, which was approximately half the size of the ChIP–seq library fragments, or to the fragment midpoint for paired-end libraries. Resulting counts per peak region were scaled by 1 × 10^−6^/total reads in each sample and multiplied by 1 × 10^−3^, then smoothed by calculating a running mean of 20 bp across the normalized counts. These were converted into normalized matrices, replicate averaged and visualized using the as.normalizedMatrix and EnrichedHeatmap functions from the EnrichedHeatmap^86^ R package. Color scales were implemented manually based on enrichment values using the colorRamp2 function within the Circlize^87^ R package. For histone mark metaprofiles, profiles from each mark were normalized by dividing by their maximum value or by 1.5 if the maximum was <1.5 (ref. ^88^). To cluster p53 peaks by enrichment for histone marks, K-means clustering on histone datasets was performed using the kmeans function from the R stats^89^ package with the following arguments: centres = 4 and nstart = 10. Publicly available datasets used in this study are as indicated in Supplementary Table 2. Browser screenshots were generated using the Gviz^90^ R package (v.1.40.1).

#### RNA sequencing

RNA sequencing (RNA-seq) reads were mapped to mm10 using STAR^91^ aligner (v.2.5.2b). Alignments were performed with otherwise default settings using the arguments –outFilterType BySJout, –outFilterMultimapNmax 20, –alignSJoverhangMin 8, –alignSJDBoverhangMin 3, –alignIntronMin 20, –outSAMmultNmax 1, allowing for up to 20 matches for a multimapping read and outputting one at random in these cases. Resulting alignment files were indexed using SAMtools^92^ (v.1.2) with default parameters. Alignments overlapping with protein-coding genes were counted using the Rsubread^93^ Bioconductor package. In brief, the featureCounts function from Rsubread was run with otherwise default settings and with the gene annotation from the M25 GENCODE^94^ release, and with GTF.attrType = ‘gene_name’ to group gene features (for example, exons). GENCODE annotations (that is, protein_coding) were used to retain only counts for protein-coding genes and to exclude predicted genes (that is, those with ID number beginning ‘Gm’). Count matrices were generated and TMM normalized (Trimmed mean of M-values) using the DGEList and calcNormFactors functions from the edgeR^95^ package with default settings. Finally, counts per million (CPM) were generated using the cpm function from edgeR, with prior.count = 8 and log = True, to add a pseudocount of 8 and to log_2_ transform the data. The statistical significance of differential expression between groups of samples was analyzed using edgeR. Briefly, read counts over protein-coding genes were generated and normalized as above for specific samples, and a design matrix was generated for these in the groups being compared using the model.matrix function. Significance and fold change estimates were generated by the estimateDisp, glmQLFit, glmQLFTest and predFC functions in the edgeR package, followed by multiple testing correction with the Benjamini–Hochberg method. Genes were considered to be differentially expressed with a false discovery rate (FDR) < 0.01 and an absolute log_2_ fold change of at least 0.75. A consensus set of Trim24 target genes in the Trim24 degron line were identified as those that were differentially expressed upon degradation of Trim24 at the 4-h timepoint of p53 activation; a later timepoint (8 h) was well correlated, and effects were largely unidirectional, that is, expression increased compared with that of Trim24-expressing cells. Genes increasing in expression at this point were enriched in Trim24 and p53 binding in promoters (*P* < 2.2 × 10^−16^; odds ratio 5.4); at the 12-h timepoint, their expression levels were less well correlated and roughly even numbers of genes further increased and decreased, probably owing to secondary effects on gene expression as a result of misexpression of the primary targets (Supplementary Fig. 12b). This consensus set therefore represents a conservative estimate of direct Trim24 target genes. To test the distance relationships between differentially expressed genes and genomic regions (that is, p53-binding peak regions), the annotatePeak function from the ChIPseeker^96^ package was used to identify genomic regions within or nearby genes. Briefly, annotatePeak was run with default arguments using the TxDb.Mmusculus.UCSC.mm10.knownGene^97^ (version 3.10.0) and org.Mm.eg.db^98^ (R package version 3.8.2) annotation packages. Functional enrichment analysis of Trim24 target genes was carried out by the R package ‘clusterProfiler’ using gene ontology and Kyoto Enyclopedia of Genes and Genomes annotations. The featureCounts tool^99^ (v.2.0.0) was used to determine RNA-seq counts over repetitive elements (for example, Lines, Sines, LTR-containing retrotransposons, etc.) using RepeatMasker^100^ annotations (v.4.1.2) in Gtf format obtained from rhw UCSC^101^ genome browser database (mm10). edgeR, as described above, was used to assess the differential expression of repetitive elements upon Trim24 degradation. To identify p53 motifs in repeat LTR elements, the Repbase^102,103^ (v.20.02) consensus sequences were scanned for the p53 weight matrix derived from the Jaspar MA0106.3 p53 motif using the matchPWM function from the Biostrings^104^ R package (version 2.68.1). Matching sequences were determined by requiring a log_2_ odds score of at least 10 over a uniform background. Browser screenshots were generated using the Gviz^90^ R package (v.1.40.1).

#### ATAC–seq

ATAC–seq reads were trimmed using cutadapt^105^ (v.2.5) with parameters -a CTGTCTCTTATACACA -A CTGTCTCTTATACACA -m 10–overlap = 1 and then mapped to mm10 using QuasR^76^ with default settings. To determine read counts over genomic regions (for example, ChIP–seq peaks), reads were first counted over all regions using the QuasR^76^ function qCount with default parameters. Counts between samples were normalized using edgeR. In brief, ATAC–seq peaks for samples being compared were first generated by MACS2 as described above for ChIP–seq and any peaks overlapping in at least two samples were retained and merged using the reduce function of the GenomicRanges^106^ R package, excluding those overlapping mm10 blacklisted regions^82^. Reads counts at merged peaks were then determined by qCount with default parameters. The resulting count data were then merged with counts over peak regions (that is, ChIP–seq peaks), and the combined set was TMM normalized using the DGEList and calcNormFactors functions from the edgeR^95^ package with default settings. Finally, CPM were generated using the cpm function from edgeR with prior.count = 8 and log = True, to add a pseudocount of 8 and to log_2_ transform the data. This ensured that counts in regions of interest (that is, ChIP–seq peaks) were normalized against all accessible chromatin sites, the majority of which were not expected to change between samples. ATAC–seq counts in ATAC–seq peaks were generated in the same manner. The statistical significance of differences in accessibility between groups of samples was determined using edgeR. Briefly, read counts over regions (that is, ATAC–seq peaks) were generated and normalized as above for specific samples and a design matrix was generated for these in the groups being compared using the model.matrix function. Significance and fold change estimates were generated using the estimateDisp, glmQLFit, glmQLFTest and predFC functions in the edgeR package. Regions were considered to be differentially accessible if they had FDR < 0.05 and fold change >0.5. The monaLisa^107^ R package was used to identify motifs that were enriched in regions that changed between samples. Briefly, fold changes between samples were generated by subtracting the replicate-averaged counts in genomic regions (that is, ATAC–seq peaks) and genomic sequence within peaks resized to the median of all peaks being considered around the peak center, using the trim and getSeq tools from the GenomicRanges^106^ and Biostrings^104^ R libraries, respectively. Fold changes between regions were binned using the bin function in the monaLisa library with default parameters and minAbsX = 1 to set the minimal absolute value for log_2_ changes, and the number of regions per changing bin was set using the nElements argument as described in each figure. Enrichment of motifs was performed with the calcBinnedMotifEnrR function, using motifs from the JASPAR2020 (ref. ^108^) database; motifs were included if they had at least −log_10_ adjusted *P* value >4 and log_2_ enriched >0.5 within bins, excluding the nonchanging center bin. For visualization, enriched motifs were clustered by similarity using the motifSimilarity and hclust tools from the monaLisa^107^ and stats^89^ packages. Heatmaps were generated by counting the midpoint positions of mapped fragments relative to defined genomic regions (that is, peak regions) using the QuasR^76^ function qProfile and visualized using the EnrichedHeatmap^86^ Bioconductor package. In brief, qProfile was run with default parameters and with the parameters shift = ‘halfInsert’ and useRead = ‘first’, for 1 kb regions centered on the middle of peak regions. Resulting counts per peak region were scaled by 1 × 10^−6^/total reads in each sample and multiplied by 1 × 10^−3^, then smoothed by calculating a running mean of 20 bp across the normalized counts. These were converted into normalized matrices, replicate averaged, and visualized using the as.normalizedMatrix and EnrichedHeatmap functions from the EnrichedHeatmap^86^ R package. Color scales were implemented manually based on enrichment values using the colorRamp2 function within the Circlize^87^ R package. Browser screenshots were generated using the Gviz^90^ R package (v.1.40.1).

#### DNase I hypersensitive sites sequencing

DNase I hypersensitive sites sequencing datasets were aligned to the mm10 mouse assembly using the QuasR^76^ Bioconductor package, which uses Bowtie^77^ (RBowtie package). Alignments were performed with the default settings, allowing for uniquely mapping reads. Read counts over defined genomic regions (that is, peak regions) were obtained using the QuasR^76^ function qCount with default parameters and shifting the reads 20 bp. Briefly, counts were normalized between datasets being compared, a pseudocount of 8 was added and data were log_2_ transformed. Normalization was performed by multiplying counts by a scaling factor that was determined by the library with the lowest number of mapped reads between the datasets, that is, scaled down to the smaller library: scaling factor χ = min(Sample 1, Sample 2,…, Sample χ)/Sample χ, where Sample 1, Sample 2,…, Sample χ are the total numbers of mapped reads in the respective samples. The pseudocount of 8 was used to decrease noise at low read counts between samples. The mean counts between replicates at genomic regions (that is, peaks) were considered, and a cut-off of >log_2_ 6 counts was used to define open and closed regions, as this reflected an inflection point in the data using qqnorm and qqline functions from the stats^89^ R package. Heatmaps were generated by counting the 5′ positions of mapped reads relative to defined genomic regions (that is, peak regions) using the QuasR^76^ function qProfile and visualized using the EnrichedHeatmap^86^ Bioconductor package. In brief, qProfile was run with default parameters for 1-kb regions centered on the middle of each peak region. Resulting counts per peak region were scaled by 1 × 10^−6^/total reads in each sample and multiplied by 1 × 10^−3^, then smoothed by calculating a running mean of 20 bp across the normalized counts. These were converted into normalized matrices, replicate averaged and visualized using the as.normalizedMatrix and EnrichedHeatmap functions from the EnrichedHeatmap^86^ R package. Color scales were implemented manually based on enrichment values using the colorRamp2 function in the Circlize^87^ R package. The publicly available human Encode datasets (that is, mapped bam files) used in this study were as indicated in Supplementary Table 2).

Quality control of sequencing data was carried out using Qualimap^109^ (v.2.2.1) with the ‘bamqc’ and ‘rnaseq’ modes. The quality of ChIP–seq, ATAC–seq and RNA-seq datasets was further assessed using the R ChIPQC^110^ package (v.1.28.0) (Supplementary Table 3).

#### Immunoprecipitation with mass spectrometry for protein enrichment analysis

Protein identification and relative quantification were performed with MaxQuant v.1.5.3.8 using Andromeda as the search engine^111^ and label-free quantification^112^. The mouse subset of UniProt v.2019_04 combined with the contaminant database from MaxQuant was searched, and the protein and peptide FDR values were set to 1% and 0.1%, respectively. The combined intensities of peptides of proteins were imported into R, and values were normalized between samples by dividing values of each sample by the sum of all values within a sample, then multiplying these by the sum of all values of the sample with the lowest sum value. Thus, values were scaled down to the sample with the lowest signal. Data were log_2_ transformed after dividing samples by 2^20^ and adding a pseudocount of 5 to stabilize the variance of the data. p53-enriched samples were compared with datasets generated by immunoprecipitation with mass spectrometry in the p53 degron line after degradation (that is, mock immunoprecipitation), and significance estimates were generated using the eBayes function in the limma^113^ R package. Briefly, the lmFit, makeContrasts and contrasts.fit tools from limma were used with default parameters to generate linear models and estimate coefficients and standard errors from samples grouped by treatment condition (that is, p53-degraded, untreated, p53-activated). Finally, adjusted *P* values were generated by the eBayes and topTable functions from limma, and proteins with adjusted *P* value < 0.01 were considered to be significantly enriched. As Fkbp1a peptides constitute the degron tag that was added to the endogenous p53 gene in this experiment, the Fkbp1a protein was manually removed from the list of enriched proteins.

#### Estimate of coefficient contributions to linear regression models

To estimate coefficient contributions to Trim24-addback binding datasets, the lm tool from the stats^89^ R package was used to generate linear regression models. These were visualized using the plot_summs function within the jtools^114^ R package (version 2.2.0). Briefly, lm was run using replicate-averaged enrichments for Trim24-addback variant ChIP–seq datasets, using default parameters and with p53 enrichment and enrichment of histone marks as independent variables. The plot_summs function was run with default parameters on the resulting fits using default parameters to visualize estimates of coefficient contributions to models.

#### Correlation heatmaps

Correlation heatmaps were generated to group effects and show reproducibility with the cor and aHeatmap functions from the stats^89^ and NMF^115^ packages. Where applicable, dataset counts, either averages across samples or individual replicates, were used within the cor function to generate Pearson correlation coefficients. These were used directly within aHeatmap, which computes a dendrogram from hierarchical clustering.

**Extended Data Fig. 3:**
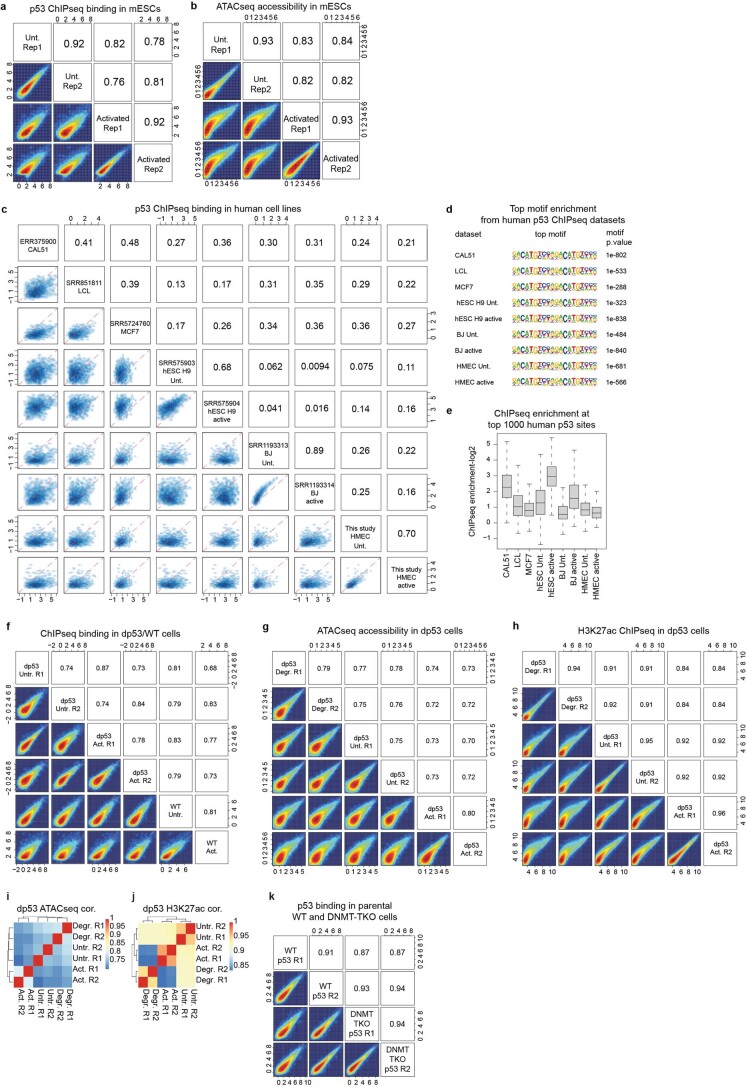
Quality control of p53-genomics datasets. (**a**) Reproducibility of p53 ChIPseq signal in mESCs, in basal and activated conditions (1uM doxorubicin, 4hrs), at the joint set of p53 peaks (n = 19259 sites). Shown are log_2_ enrichments over IgG controls for independent replicates (Rep1/Rep2). Pearson correlation coefficients indicated. (**b**) ATACseq signal reproducibility in mESCS (log_2_ CPM) basal and activated conditions, at p53 peaks (n = 19259 sites). At least half of all peaks (n = 9781) show increased accessibility upon activation, that is, ≥2-fold increase. Pearson correlation coefficients indicated. (**c**) Log_2_ enrichments of p53 ChIPseq signal over control datasets in various human cell lines. Publicly available datasets are as indicated, as well as cell line description and the p53-induction state per study-conditions. Data shown at the top 1000 p53 peaks, as ranked by mean signal across datasets, and represent sites that are commonly bound. Pearson correlation coefficients indicated. (**d**) Top known motifs enriched from p53 binding datasets by HOMER. (**e**) Boxplot showing log_2_ ChIPseq enrichments as in **c**, using data from single replicate experiments. Centre median to first/third quartile, whiskers to 1.5 multiplied by interquartile range. (**f**) Reproducibility of mESC dp53 ChIPseq signal, in basal and activated conditions alongside average enrichment of WT p53, at p53 peaks (n = 19259 sites). Shown are log_2_ enrichments over IgG control datasets for replicates (R1/R2). Pearson correlation coefficients indicated. (**g**) ATACseq signal reproducibility (log_2_ CPM) in *dp53* mESCs, in degraded, basal and activated conditions, for replicates (R1/R2) at p53 peaks (n = 19259 sites). Pearson correlation coefficients indicated. (**h**) Reproducibility of H3K27ac ChIPseq (log_2_ normalized signal) in dp53 mESCs under degraded, basal and activated conditions for replicates (R1/R2), at p53 peaks (n = 19259 sites). Pearson correlation coefficients indicated. (**i**) Correlation-based clustering of ATACseq signal or (**j**) H3K27ac ChIPseq signal in the *dp53*mESCs. Pearson correlations on log_2_ counts indicated. **k**) Reproducibility of p53 ChIPseq signal in DNMT-TKOs, and parental WT line, in basal conditions. Shown are log_2_ enrichments over IgG controls for replicates (R1/R2) at p53 peaks (n = 19259 sites). Pearson correlation coefficients indicated.

**Extended Data Fig. 4:**
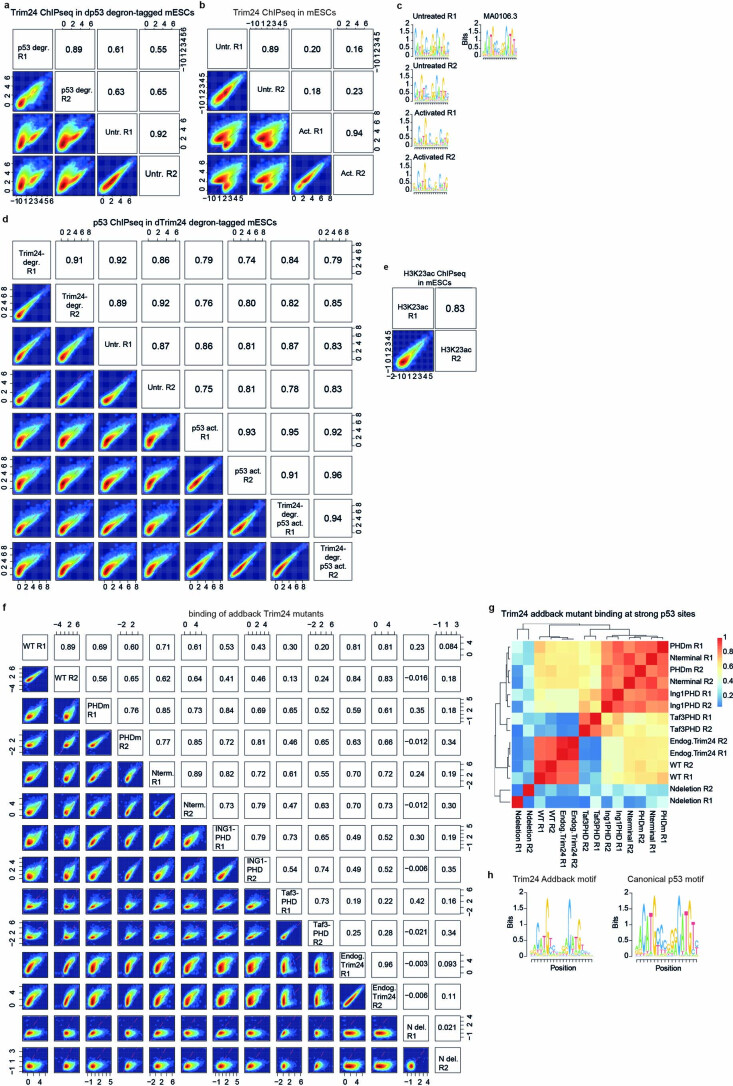
Quality control of Trim24-genomics datasets. (**a**) Reproducibility of Trim24 ChIPseq signal in the dp53 degron-tag line, in basal and p53-degraded conditions, at all Trim24 peaks (n = 25768 sites). Shown are log_2_ enrichments over IgG control datasets for independent replicates (R1/R2). Pearson correlation coefficients are indicated. (**b**) Reproducibility of Trim24 ChIPseq signal in mESCs, in basal and p53-activated conditions, at the joint set of Trim24 peaks (n = 25768 sites). Shown are log_2_ enrichments over IgG control datasets for replicates (R1/R2). Pearson correlation coefficients are indicated. (**c**) Top *de novo* HOMER motifs enriched from independent Trim24 ChIPseq experiments from **c**, reminiscent of the canonical p53 motif (below, Jaspar motif MA0106.3). (**d**) Reproducibility of p53 ChIPseq signal in a dTrim24 degron-tag line, in basal and p53-activated conditions and upon Trim24 degradation, at the combined set of Trim24 and p53 peaks (n = 29871). Shown are log_2_ enrichments over IgG control datasets for independent replicates (R1/R2). Pearson correlation coefficients are indicated. (**e**) Reproducibility of H3K23ac ChIPseq signal in mESCs at the joint set of p53 and Trim24 peaks in p53-active conditions (n = 23677 sites). Shown are log_2_ enrichments over IgG control datasets for independent replicates (R1/R2). Pearson correlation coefficients are indicated. (**f**) Reproducibility of Flag-ChIPseq signal in addback-Trim24 expressing cells at the joint set of addback-Trim24 peaks in p53-active conditions (n = 30542 sites), as well as endogenous Trim24 ChIPseq. Shown are log_2_ enrichments over IgG control datasets for independent replicates (R1/R2). Pearson correlation coefficients are indicated. (**g**) Correlation-based clustering of addback-Trim24 samples at strong p53 binding sites (n = 5804 sites, ≥ 3.5-fold log_2_ p53 ChIPseq enrichment). Pearson correlations are as indicated. (**h**) Top *de novo* HOMER motif enriched from addback-Trim24 peaks, strongly reminiscent of the canonical p53 motif (right, Jaspar motif MA0106.3).

**Extended Data Fig. 5:**
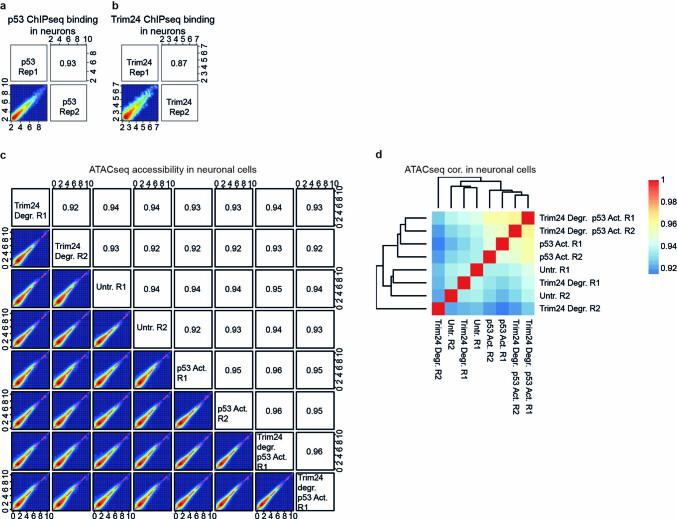
Quality control of neuronal genomics datasets. (**a**) Reproducibility of p53 ChIPseq signal in the neuronal cells differentiated from the Trim24 degron line, in p53 active conditions, at all neuronal p53 peaks (n = 9535 sites). Shown are log_2_ enrichments over IgG control datasets for independent replicates (R1/R2). Pearson correlation coefficients are indicated. (**b**) Reproducibility of Trim24 ChIPseq signal in the neuronal cells differentiated from the Trim24 degron line, in p53 active conditions, at all neuronal Trim24 peaks (n = 2325 sites). Shown are log_2_ enrichments over IgG control datasets for independent replicates (R1/R2). Pearson correlation coefficients are indicated. (**c**) Reproducibility of ATACseq signal (log_2_ CPM) in the neuronal cells differentiated from the Trim24 degron line, in degraded, untreated and activated conditions with or without Trim24 degradation, for replicates (R1/R2) at all neuronal ATACseq peaks (n = 62070 sites). Pearson correlation coefficients are indicated. (**d**) Correlation-based clustering of ATACseq signal in the neuronal cells differentiated from the Trim24 degron line. Pearson correlations on log_2_ counts are as indicated.

To demonstrate the reproducibility of replicate ChIP–seq and ATAC–seq experiments, quality control plots (for example, scatter plots and correlation heatmaps) were generated from datasets to demonstrate agreement in the raw signal at regions of interest in the genome (that is, dataset peak regions) (Extended Data Figs. 3–5).

In all box plots, middle points correspond to median, boxes to first and third quartile, and whiskers to 1.5 multiplied by the interquartile range. Notches, where indicated, extend to ±1.58 × (interquartile range/square_root(*n*)). Whiskers correspond to the maximum and minimum distribution values after removal of outliers, where outliers are defined as more than 1.5 × (interquartile range) away from the box. Pearson correlation coefficients were calculated using the R function cor with default parameters.

For ChIP–seq, Illumina RTA 1.18.64 and bcl2fastq2 v.2.17 were used for basecalling and demultiplexing for single-read experiments, and Illumina RTA 2.4.11 and bcl2fastq2 v.2.17 were used for basecalling and demultiplexing for paired-read experiments. For RNA-seq, Illumina RTA 1.18.64 and bcl2fastq2 v.2.17 were used for basecalling and demultiplexing samples generated by Illumina HiSeq sequencing. Illumina RTA 3.4.4 and bcl2fastq2 v.2.20 were used for basecalling and demultiplexing samples generated by Illumina NovaSeq sequencing. For ATAC–seq, Illumina RTA 2.4.11 and bcl2fastq2 v.2.17 were used for basecalling and demultiplexing.

#### Statistics and reproducibility

All experiments in this study for which selected images are shown, including representative microscopy images and gel blot images selected during standard cumulative exposure capture, were single instance experiments and represent unique data collection events. Images were selected for display features and otherwise at random.

### Reporting summary

Further information on research design is available in the Nature Portfolio Reporting Summary linked to this article.

## Online content

Any methods, additional references, Nature Portfolio reporting summaries, source data, extended data, supplementary information, acknowledgements, peer review information; details of author contributions and competing interests; and statements of data and code availability are available at 10.1038/s41594-023-01021-8.

## Supplementary information

Supplementary Information Supplementary Figs. 1–16, inventory of source data files, legends for Tables 1–3 and legends for Videos 1 and 2.

Reporting Summary

Supplementary Tables Supplementary Tables 1–3.

Supplementary Video 1 Supplementary Video 1.

Supplementary Video 2 Supplementary Video 2.

## Data Availability

Next-generation sequencing data are available via the Gene Expression Omnibus, accession number GSE200586. The mass spectrometry proteomics data have been deposited to the ProteomeXchange Consortium via the PRIDE^116^ partner repository with the dataset identifiers PXD033674 and PXD039553. Source data are provided with this paper.

## Source data

Source Data for Fig. 2 Raw western blot images for Fig. 2.
Source Data for Fig. 4 Raw western blot images for Fig. 4.
