# Supplementary material for: Readout of histone methylation by Trim24 locally restricts chromatin opening by p53
Source: Nat Struct Mol Biol. 2023 Jun 29;30(7):948–57. doi: 10.1038/s41594-023-01021-8 (PMC10352137; doi:10.1038/s41594-023-01021-8)
Supplement: Supplementary file 2 — Reporting Summary [file 41594_2023_1021_MOESM2_ESM.pdf]

nature portfolio

Corresponding author(s): Dirk Schübeler

Last updated by author(s): 1/01/2023

Reporting Summary

Nature Portfolio wishes to improve the reproducibility of the work that we publish. This form provides structure for consistency and transparency in reporting. For further information on Nature Portfolio policies, see our Editorial Policies and the Editorial Policy Checklist.

Statistics

For all statistical analyses, confirm that the following items are present in the figure legend, table legend, main text, or Methods section.

n/a	Confirmed	
☐	☒	The exact sample size (*n*) for each experimental group/condition, given as a discrete number and unit of measurement
☐	☒	A statement on whether measurements were taken from distinct samples or whether the same sample was measured repeatedly
☐	☒	The statistical test(s) used AND whether they are one- or two-sided<br>*Only common tests should be described solely by name; describe more complex techniques in the Methods section.*
☐	☒	A description of all covariates tested
☐	☒	A description of any assumptions or corrections, such as tests of normality and adjustment for multiple comparisons
☐	☒	A full description of the statistical parameters including central tendency (e.g. means) or other basic estimates (e.g. regression coefficient) AND variation (e.g. standard deviation) or associated estimates of uncertainty (e.g. confidence intervals)
☐	☒	For null hypothesis testing, the test statistic (e.g. *F*, *t*, *r*) with confidence intervals, effect sizes, degrees of freedom and *P* value noted<br>*Give P values as exact values whenever suitable.*
☒	☐	For Bayesian analysis, information on the choice of priors and Markov chain Monte Carlo settings
☒	☐	For hierarchical and complex designs, identification of the appropriate level for tests and full reporting of outcomes
☐	☒	Estimates of effect sizes (e.g. Cohen's *d*, Pearson's *r*), indicating how they were calculated

Our web collection on statistics for biologists contains articles on many of the points above.

Software and code

Policy information about availability of computer code

Data collection	Software used for data collection: For ChIPseq, Illumina RTA 1.18.64 and bcl2fastq2 v2.17 was used for basecalling and demultiplexing for single-read experiments, Illumina RTA 2.4.11 and bcl2fastq2 v2.17 was used for basecalling and demultiplexing for paired-read experiments. For RNAseq, Illumina RTA 1.18.64 and bcl2fastq2 v2.17 was used for basecalling and demultiplexing samples generated by Illumina HiSeq sequencing. Illumina RTA 3.4.4 and bcl2fastq2 v2.20 was used for basecalling and demultiplexing samples generated by Illumina NovaSeq sequencing. For ATACseq, Illumina RTA 2.4.11 and bcl2fastq2 v2.17 was used for basecalling and demultiplexing. Protein identification and relative quantification was performed with MaxQuant v.1.5.3.8.
Data analysis	Software used in the study: MACS2 peak caller (version 2.1.3.3), bedtools (version 2.25.0), GraphPad Prism (version 8), Homer motif finder (version 4.8), ATAC-seq read were trimmed using cutadapt 2.5, RNAseq reads were mapped to mm10 using STAR (version 2.5.2b). Data analysis was performed in R (4.2) and using BSgenome.Mmusculus.UCSC.mm10 1.4.0, BSgenome.Hsapiens.UCSC.hg38 1.4.1, TxDb.Mmusculus.UCSC.mm10.knownGene 3.4.4, ATACseq and ChIPseq were mapped using QuasR 1.26.0 which implements Rbowtie 1.26.0, DiffBind 3.2.4, org.Mm.eg.db 3.15.0, featureCounts 2.0.0, RepeatMasker 4.1.2, edgeR 3.38.1, Repbase 20.02, Gviz 1.40.1, ChIPseeker 1.28.3, Biostrings 2.54.0, GenomicRanges 1.48.0, ImageJ, GraphPad Prism 8 , Microsoft Excel version 2303, SeqLog 1.64.0, EnrichedHeatmap 1.26.0, monaLisa 1.4.0, stats R package 4.2.2, NMF 0.26, Samtools 1.2, Rsubread 2.12.3.

For manuscripts utilizing custom algorithms or software that are central to the research but not yet described in published literature, software must be made available to editors and reviewers. We strongly encourage code deposition in a community repository (e.g. GitHub). See the Nature Portfolio guidelines for submitting code & software for further information.

Data

Policy information about availability of data

All manuscripts must include a data availability statement. This statement should provide the following information, where applicable:

- Accession codes, unique identifiers, or web links for publicly available datasets
- A description of any restrictions on data availability
- For clinical datasets or third party data, please ensure that the statement adheres to our policy

Next-generation sequencing data are available via the Gene Expression Omnibus, accession number GSE200586.
The mass spectrometry proteomics data have been deposited to the ProteomeXchange Consortium via the PRIDE partner repository with the dataset identifier PXD033674 and PXD039553.

Human research participants

Policy information about studies involving human research participants and Sex and Gender in Research.

Reporting on sex and gender	n/a
Population characteristics	n/a
Recruitment	n/a
Ethics oversight	n/a

Note that full information on the approval of the study protocol must also be provided in the manuscript.

Field-specific reporting

Please select the one below that is the best fit for your research. If you are not sure, read the appropriate sections before making your selection.

☒ Life sciences ☐ Behavioural & social sciences ☐ Ecological, evolutionary & environmental sciences

For a reference copy of the document with all sections, see nature.com/documents/nr-reporting-summary-flat.pdf

Life sciences study design

All studies must disclose on these points even when the disclosure is negative.

Sample size	For all genomics experiments where a delta was examined (i.e. ChIPseq, ATACseq, RNAseq et), samples were produced in at least duplicate to query the reproducibility and where appropriate, the statistical significance of the effect size. In particular, gene expression changes were produced in at least triplicate to ensure statistical tests could be carried out with at least three replicates. Microscopy were performed with at least 100 cells/measure to ensure trends were sufficiently measured and where appropriate (i.e. time lapse imaging data) at least 6 planes were selected for sampling to ensure reproducibility. Additional experimental data points (i.e.)alpha counts) were performed in duplicate to ensure reproducibility of effects. Cell viability measurements were performed in triplicate to ensure statistical tests could be carried out with at least three replicates. Gel blots and Westerns were performed with single replicates which are sufficient to observe large trends individually.
Data exclusions	No data were excluded from the study.
Replication	The number of replicates per experiment are as indicated in the study, all experimental data that require direct comparison (i.e. changes in NGS signal, change in cell density, change in alpha counts, change in protein abundance) were generated from batch-experiments where samples were generated in paralele at the same time and with the same reagents. All attempts at replication were successful.
Randomization	The study conditions did not require randomization as this study design is not affected by the order of recording for experimental data.
Blinding	The study conditions did not require blinding as this study design is not affected by the observer carrying out the experiments.

Reporting for specific materials, systems and methods

We require information from authors about some types of materials, experimental systems and methods used in many studies. Here, indicate whether each material, system or method listed is relevant to your study. If you are not sure if a list item applies to your research, read the appropriate section before selecting a response.

Materials & experimental systems

n/a	Involved in the study
☐	☒ Antibodies
☐	☒ Eukaryotic cell lines
☒	☐ Palaeontology and archaeology
☒	☐ Animals and other organisms
☒	☐ Clinical data
☒	☐ Dual use research of concern

Methods

n/a	Involved in the study
☐	☒ ChIP-seq
☒	☐ Flow cytometry
☒	☐ MRI-based neuroimaging

Antibodies

Antibodies used	p53-2524-Cell signaling technology, p53-S15phosphorylation-9284-Cell signaling technology, Flag-F1804-Sigma Aldrich, Actin-B-8H10D10-Cell signaling technology, Lamin-B1-ab16048-Abcam, COX2-ab15191-Abcam, Trim24-14208-1-AP-Proteintech, Trim24-A300-815A-Bethyl laboratories, V5-R96025-Thermo Fisher Scientific, V5-MA5-32053-Thermo Fisher Scientific, IgG-M7023-Sigma Aldrich, H3K23ac-39131-Active Motif, H3K27ac-39133-Active Motif, FLAG-F1804-Sigma Aldrich, anti-rabbit-IgG HRP -NA-934-1ml GE Healthcare, anti-mouse-IgG HRP -NA931-1ml GE Healthcare
Validation	all antibodies were validated by the manufacturer. In detail: p53-2524 validated in human cell line extracts - Western and immunofluorescent signal and in ChIPqPCR. p53-S15phosphorylation-9284 validated in human cell line extracts - Western and in ChIPqPCR. Flag-F1804 validated to work in mammalian cells by the manufacturer for techniques including Western blotting, immunofluorescence, and immunoprecipitation. Actin-B-8H10D10 validated in human cell line extracts - Western and immunofluorescent signal. Lamin-B1-ab16048 validated in human cell line extracts - Western and immunofluorescent signal. COX2-ab15191 validated to work in mouse and human tissue for immunofluorescent signal. Trim24-14208-1-AP validated in human cell line extracts and human and mouse tissue extracts - Western, as well as human tissue for immunofluorescent signal. Trim24-A300-815A validated in human and mouse cell extracts - Western and human tissue for immunofluorescent signal. V5-R96025 validated in human cell line extracts - Western and immunofluorescent signal. V5-MA5-32053 validated in human cell line extracts - Western and immunofluorescent signal. IgG-M7023-Sigma Aldrich validated in mouse tissue by immunofluorescent signal. H3K23ac-39131 validated in human cell line extracts - Western and immunofluorescent signal and in ChIPqPCR.

Eukaryotic cell lines

Policy information about cell lines and Sex and Gender in Research

Cell line source(s)	mouse ES cell line TC-1 were originally obtained from Dr. Ann Dean at the NIH and modified with the RMCE landing pad as described in PMID: 21964573. The DNMT triple knockout cell line was generated from this line and is described in PMID: 30709850. The p53 and Trim24 knockout and dTAG lines, Ngn2-induction and Trim24 addback and GFP expressing lines were generated in these isogenic backgrounds and are available upon request. Human Mammary Epithelial Cells (CC-2551) were obtained from Lonza. Sf9 were perchased from Thermo Fischer Scientific (Cat# 11496-015).
Authentication	Genotype of cell lines was tested at the level of DNA sequence and protein (Western blotting).
Mycoplasma contamination	Cell lines tested negative for Mycoplasm.
Commonly misidentified lines (See ICLAC register)	No commonly misidentified cell lines were used.

ChIP-seq

Data deposition

☒ Confirm that both raw and final processed data have been deposited in a public database such as GEO.

☒ Confirm that you have deposited or provided access to graph files (e.g. BED files) for the called peaks.

Data access links<br>*May remain private before publication.*	https://www.ncbi.nlm.nih.gov/geo/query/acc.cgi?acc=GSE200586 (all data including ChIPseq)
Files in database submission	For each ChIP-seq sample, the GEO entry contains the following files: the rawdata (fastq format) and a file with alignment density per 100 bp in the mouse mm10 or human hg19 genome (wig file):<br>fastq files:<br>ChIPseq_ESC_wt_p53_Unt_r1_1.fastq.gz<br>ChIPseq_ESC_wt_p53_Unt_r1_2.fastq.gz<br>ChIPseq_ESC_wt_p53_Unt_r2_1.fastq.gz<br>ChIPseq_ESC_wt_p53_Unt_r2_2.fastq.gz<br>ChIPseq_ESC_wt_p53_Act_r1_1.fastq.gz

ChIPseq_ESC_wt_p53_Act_r1_2.fastq.gz
ChIPseq_ESC_wt_p53_Act_r2_1.fastq.gz
ChIPseq_ESC_wt_p53_Act_r2_2.fastq.gz
ChIPseq_ESC_wt_Trim24_Unt_r1_1.fastq.gz
ChIPseq_ESC_wt_Trim24_Unt_r1_2.fastq.gz
ChIPseq_ESC_wt_Trim24_Unt_r2_1.fastq.gz
ChIPseq_ESC_wt_Trim24_Unt_r2_2.fastq.gz
ChIPseq_ESC_wt_Trim24_Act_r1_1.fastq.gz
ChIPseq_ESC_wt_Trim24_Act_r1_2.fastq.gz
ChIPseq_ESC_wt_Trim24_Act_r2_1.fastq.gz
ChIPseq_ESC_wt_Trim24_Act_r2_2.fastq.gz
ChIPseq_ESC_wt_IgG_Unt_r1_1.fastq.gz
ChIPseq_ESC_wt_IgG_Unt_r1_2.fastq.gz
ChIPseq_ESC_wt_IgG_Unt_r2_1.fastq.gz
ChIPseq_ESC_wt_IgG_Unt_r2_2.fastq.gz
ChIPseq_ESC_wt_H3K23ac_Unt_r1_1.fastq.gz
ChIPseq_ESC_wt_H3K23ac_Unt_r1_2.fastq.gz
ChIPseq_ESC_wt_H3K23ac_Unt_r2_1.fastq.gz
ChIPseq_ESC_wt_H3K23ac_Unt_r2_2.fastq.gz
ChIPseq_ESC_parental_p53_r1_1.fastq.gz
ChIPseq_ESC_parental_p53_r1_2.fastq.gz
ChIPseq_ESC_parental_p53_r1_3.fastq.gz
ChIPseq_ESC_parental_p53_r2_1.fastq.gz
ChIPseq_ESC_parental_p53_r2_2.fastq.gz
ChIPseq_ESC_parental_p53_r2_3.fastq.gz
ChIPseq_ESC_DNMT.TKO_p53_r1_1.fastq.gz
ChIPseq_ESC_DNMT.TKO_p53_r1_2.fastq.gz
ChIPseq_ESC_DNMT.TKO_p53_r1_3.fastq.gz
ChIPseq_ESC_DNMT.TKO_p53_r2_1.fastq.gz
ChIPseq_ESC_DNMT.TKO_p53_r2_2.fastq.gz
ChIPseq_ESC_DNMT.TKO_p53_r2_3.fastq.gz
ChIPseq_ESC_dTrim24_p53_Degr_r1_1.fastq.gz
ChIPseq_ESC_dTrim24_p53_Degr_r1_2.fastq.gz
ChIPseq_ESC_dTrim24_p53_Degr_r2_1.fastq.gz
ChIPseq_ESC_dTrim24_p53_Degr_r2_2.fastq.gz
ChIPseq_ESC_dTrim24_p53_Unt_r1_1.fastq.gz
ChIPseq_ESC_dTrim24_p53_Unt_r1_2.fastq.gz
ChIPseq_ESC_dTrim24_p53_Unt_r2_1.fastq.gz
ChIPseq_ESC_dTrim24_p53_Unt_r2_2.fastq.gz
ChIPseq_ESC_dTrim24_p53_Act_r1_1.fastq.gz
ChIPseq_ESC_dTrim24_p53_Act_r1_2.fastq.gz
ChIPseq_ESC_dTrim24_p53_Act_r2_1.fastq.gz
ChIPseq_ESC_dTrim24_p53_Act_r2_2.fastq.gz
ChIPseq_ESC_dTrim24_p53_DegrAct_r1_1.fastq.gz
ChIPseq_ESC_dTrim24_p53_DegrAct_r1_2.fastq.gz
ChIPseq_ESC_dTrim24_p53_DegrAct_r2_1.fastq.gz
ChIPseq_ESC_dTrim24_p53_DegrAct_r2_2.fastq.gz
ChIPseq_ESC_dTrim24_IgG_Unt_r1_1.fastq.gz
ChIPseq_ESC_dTrim24_IgG_Unt_r1_2.fastq.gz
ChIPseq_ESC_dTrim24_IgG_Unt_r2_1.fastq.gz
ChIPseq_ESC_dTrim24_IgG_Unt_r2_2.fastq.gz
ChIPseq_ESC_dp53_p53_Unt_r1_1_read1.fastq.gz
ChIPseq_ESC_dp53_p53_Unt_r1_1_read2.fastq.gz
ChIPseq_ESC_dp53_p53_Unt_r2_1_read1.fastq.gz
ChIPseq_ESC_dp53_p53_Unt_r2_1_read2.fastq.gz
ChIPseq_ESC_dp53_p53_Act_r1_1_read1.fastq.gz
ChIPseq_ESC_dp53_p53_Act_r1_1_read2.fastq.gz
ChIPseq_ESC_dp53_p53_Act_r2_1_read1.fastq.gz
ChIPseq_ESC_dp53_p53_Act_r2_1_read2.fastq.gz
ChIPseq_ESC_dp53_IgG_Unt_r1_1_read1.fastq.gz
ChIPseq_ESC_dp53_IgG_Unt_r1_1_read2.fastq.gz
ChIPseq_ESC_dp53_IgG_Unt_r2_1_read1.fastq.gz
ChIPseq_ESC_dp53_IgG_Unt_r2_1_read2.fastq.gz
ChIPseq_ESC_dp53_Trim24_Degr_r1_1.fastq.gz
ChIPseq_ESC_dp53_Trim24_Degr_r1_2.fastq.gz
ChIPseq_ESC_dp53_Trim24_Degr_r1_3.fastq.gz
ChIPseq_ESC_dp53_Trim24_Degr_r2_1.fastq.gz
ChIPseq_ESC_dp53_Trim24_Degr_r2_2.fastq.gz
ChIPseq_ESC_dp53_Trim24_Degr_r2_3.fastq.gz
ChIPseq_ESC_dp53_Trim24_Unt_r1_1.fastq.gz
ChIPseq_ESC_dp53_Trim24_Unt_r1_2.fastq.gz
ChIPseq_ESC_dp53_Trim24_Unt_r1_3.fastq.gz
ChIPseq_ESC_dp53_Trim24_Unt_r2_1.fastq.gz
ChIPseq_ESC_dp53_Trim24_Unt_r2_2.fastq.gz
ChIPseq_ESC_dp53_Trim24_Unt_r2_3.fastq.gz
ChIPseq_ESC_dp53_H3K27ac_Degr_r1_1_read1.fastq.gz

ChIPseq_ESC_dp53_H3K27ac_Degr_r1_1_read2.fastq.gz
ChIPseq_ESC_dp53_H3K27ac_Degr_r1_2_read1.fastq.gz
ChIPseq_ESC_dp53_H3K27ac_Degr_r2_1_read1.fastq.gz
ChIPseq_ESC_dp53_H3K27ac_Degr_r2_1_read2.fastq.gz
ChIPseq_ESC_dp53_H3K27ac_Degr_r2_2_read1.fastq.gz
ChIPseq_ESC_dp53_H3K27ac_Unt_r1_1_read1.fastq.gz
ChIPseq_ESC_dp53_H3K27ac_Unt_r1_1_read2.fastq.gz
ChIPseq_ESC_dp53_H3K27ac_Unt_r1_2_read1.fastq.gz
ChIPseq_ESC_dp53_H3K27ac_Unt_r2_1_read1.fastq.gz
ChIPseq_ESC_dp53_H3K27ac_Unt_r2_1_read2.fastq.gz
ChIPseq_ESC_dp53_H3K27ac_Unt_r2_2_read1.fastq.gz
ChIPseq_ESC_dp53_H3K27ac_Act_r1_1_read1.fastq.gz
ChIPseq_ESC_dp53_H3K27ac_Act_r1_1_read2.fastq.gz
ChIPseq_ESC_dp53_H3K27ac_Act_r1_2_read1.fastq.gz
ChIPseq_ESC_dp53_H3K27ac_Act_r2_1_read1.fastq.gz
ChIPseq_ESC_dp53_H3K27ac_Act_r2_1_read2.fastq.gz
ChIPseq_ESC_dp53_H3K27ac_Act_r2_2_read1.fastq.gz
ChIPseq_ESC_Trim24adbkWT_Flag_Act_r1_1.fastq.gz
ChIPseq_ESC_Trim24adbkWT_Flag_Act_r2_1.fastq.gz
ChIPseq_ESC_Trim24adbkNterminal_Flag_Act_r1_1.fastq.gz
ChIPseq_ESC_Trim24adbkNterminal_Flag_Act_r1_2.fastq.gz
ChIPseq_ESC_Trim24adbkNterminal_Flag_Act_r2_1.fastq.gz
ChIPseq_ESC_Trim24adbkNterminal_Flag_Act_r2_2.fastq.gz
ChIPseq_ESC_Trim24adbkNdeletion_Flag_Act_r1_1.fastq.gz
ChIPseq_ESC_Trim24adbkNdeletion_Flag_Act_r1_2.fastq.gz
ChIPseq_ESC_Trim24adbkNdeletion_Flag_Act_r2_1.fastq.gz
ChIPseq_ESC_Trim24adbkNdeletion_Flag_Act_r2_2.fastq.gz
ChIPseq_ESC_Trim24adbkPHDmutant_Flag_Act_r1_1.fastq.gz
ChIPseq_ESC_Trim24adbkPHDmutant_Flag_Act_r2_1.fastq.gz
ChIPseq_ESC_Trim24adbkING1.PHDmutant_Flag_Act_r1_1.fastq.gz
ChIPseq_ESC_Trim24adbkING1.PHDmutant_Flag_Act_r1_2.fastq.gz
ChIPseq_ESC_Trim24adbkING1.PHDmutant_Flag_Act_r2_1.fastq.gz
ChIPseq_ESC_Trim24adbkING1.PHDmutant_Flag_Act_r2_2.fastq.gz
ChIPseq_ESC_Trim24adbkTaf3.PHDmutant_Flag_Act_r1_1.fastq.gz
ChIPseq_ESC_Trim24adbkTaf3.PHDmutant_Flag_Act_r1_2.fastq.gz
ChIPseq_ESC_Trim24adbkTaf3.PHDmutant_Flag_Act_r2_1.fastq.gz
ChIPseq_ESC_Trim24adbkTaf3.PHDmutant_Flag_Act_r2_2.fastq.gz
ChIPseq_ESC_Trim24adbkWT_IgG_Act_r1_1.fastq.gz
ChIPseq_ESC_Trim24adbkWT_IgG_Act_r2_1.fastq.gz
ChIPseq_ESC_Trim24adbkWT_IgG_Act_r2_2.fastq.gz
ChIPseq_HMEC_wt_p53_Unt_r1_1.fastq.gz
ChIPseq_HMEC_wt_p53_Unt_r1_2.fastq.gz
ChIPseq_HMEC_wt_p53_Act_r1_1.fastq.gz
ChIPseq_HMEC_wt_p53_Act_r1_2.fastq.gz
ChIPseq_HMEC_wt_IgG_Unt_r1_1.fastq.gz
ChIPseq_HMEC_wt_IgG_Unt_r1_2.fastq.gz
ChIPseq_Neuron_dTrim24_p53_Act_r1_1_read1.fastq.gz
ChIPseq_Neuron_dTrim24_p53_Act_r1_1_read2.fastq.gz
ChIPseq_Neuron_dTrim24_p53_Act_r2_1_read1.fastq.gz
ChIPseq_Neuron_dTrim24_p53_Act_r2_1_read2.fastq.gz
ChIPseq_Neuron_dTrim24_Trim24_Act_r1_1_read1.fastq.gz
ChIPseq_Neuron_dTrim24_Trim24_Act_r1_1_read2.fastq.gz
ChIPseq_Neuron_dTrim24_Trim24_Act_r2_1_read1.fastq.gz
ChIPseq_Neuron_dTrim24_Trim24_Act_r2_1_read2.fastq.gz
ChIPseq_Neuron_dTrim24_IgG_Act_r1_1_read1.fastq.gz
ChIPseq_Neuron_dTrim24_IgG_Act_r1_1_read2.fastq.gz
ChIPseq_Neuron_dTrim24_IgG_Act_r2_1_read1.fastq.gz
ChIPseq_Neuron_dTrim24_IgG_Act_r2_1_read2.fastq.gz
ChIPseq_ESC_Trim24adbkRINGmutant_Flag_Act_r1_1.fastq.gz
ChIPseq_ESC_Trim24adbkRINGmutant_Flag_Act_r1_2.fastq.gz
ChIPseq_ESC_Trim24adbkRINGmutant_Flag_Act_r2_1.fastq.gz
ChIPseq_ESC_Trim24adbkRINGmutant_Flag_Act_r2_2.fastq.gz

Wig files:
ChIPseq_ESC_wt_p53_Unt_r1.wig.gz
ChIPseq_ESC_wt_p53_Unt_r2.wig.gz
ChIPseq_ESC_wt_p53_Act_r1.wig.gz
ChIPseq_ESC_wt_p53_Act_r2.wig.gz
ChIPseq_ESC_wt_Trim24_Unt_r1.wig.gz
ChIPseq_ESC_wt_Trim24_Unt_r2.wig.gz
ChIPseq_ESC_wt_Trim24_Act_r1.wig.gz
ChIPseq_ESC_wt_Trim24_Act_r2.wig.gz
ChIPseq_ESC_wt_IgG_Unt_r1.wig.gz
ChIPseq_ESC_wt_IgG_Unt_r2.wig.gz

ChIPseq_ESC_wt_H3K23ac_Unt_r1.wig.gz
ChIPseq_ESC_wt_H3K23ac_Unt_r2.wig.gz
ChIPseq_ESC_parental_p53_r1.wig.gz
ChIPseq_ESC_parental_p53_r2.wig.gz
ChIPseq_ESC_DNMT.TKO_p53_r1.wig.gz
ChIPseq_ESC_DNMT.TKO_p53_r2.wig.gz
ChIPseq_ESC_dTrim24_p53_Degr_r1.wig.gz
ChIPseq_ESC_dTrim24_p53_Degr_r2.wig.gz
ChIPseq_ESC_dTrim24_p53_Unt_r1.wig.gz
ChIPseq_ESC_dTrim24_p53_Unt_r2.wig.gz
ChIPseq_ESC_dTrim24_p53_Act_r1.wig.gz
ChIPseq_ESC_dTrim24_p53_Act_r2.wig.gz
ChIPseq_ESC_dTrim24_p53_DegrAct_r1.wig.gz
ChIPseq_ESC_dTrim24_p53_DegrAct_r2.wig.gz
ChIPseq_ESC_dTrim24_IgG_Unt_r1.wig.gz
ChIPseq_ESC_dTrim24_IgG_Unt_r2.wig.gz
ChIPseq_ESC_dp53_p53_Unt_r1.wig.gz
ChIPseq_ESC_dp53_p53_Unt_r2.wig.gz
ChIPseq_ESC_dp53_p53_Act_r1.wig.gz
ChIPseq_ESC_dp53_p53_Act_r2.wig.gz
ChIPseq_ESC_dp53_IgG_Unt_r1.wig.gz
ChIPseq_ESC_dp53_IgG_Unt_r2.wig.gz
ChIPseq_ESC_dp53_Trim24_Degr_r1.wig.gz
ChIPseq_ESC_dp53_Trim24_Degr_r2.wig.gz
ChIPseq_ESC_dp53_Trim24_Unt_r1.wig.gz
ChIPseq_ESC_dp53_Trim24_Unt_r2.wig.gz
ChIPseq_ESC_dp53_H3K27ac_Degr_r1.wig.gz
ChIPseq_ESC_dp53_H3K27ac_Degr_r2.wig.gz
ChIPseq_ESC_dp53_H3K27ac_Unt_r1.wig.gz
ChIPseq_ESC_dp53_H3K27ac_Unt_r2.wig.gz
ChIPseq_ESC_dp53_H3K27ac_Act_r1.wig.gz
ChIPseq_ESC_dp53_H3K27ac_Act_r2.wig.gz
ChIPseq_ESC_Trim24adbkWT_Flag_Act_r1.wig.gz
ChIPseq_ESC_Trim24adbkWT_Flag_Act_r2.wig.gz
ChIPseq_ESC_Trim24adbkNterminal_Flag_Act_r1.wig.gz
ChIPseq_ESC_Trim24adbkNterminal_Flag_Act_r2.wig.gz
ChIPseq_ESC_Trim24adbkNdeletion_Flag_Act_r1.wig.gz
ChIPseq_ESC_Trim24adbkNdeletion_Flag_Act_r2.wig.gz
ChIPseq_ESC_Trim24adbkPHDmutant_Flag_Act_r1.wig.gz
ChIPseq_ESC_Trim24adbkPHDmutant_Flag_Act_r2.wig.gz
ChIPseq_ESC_Trim24adbkING1.PHDmutant_Flag_Act_r1.wig.gz
ChIPseq_ESC_Trim24adbkING1.PHDmutant_Flag_Act_r2.wig.gz
ChIPseq_ESC_Trim24adbkTaf3.PHDmutant_Flag_Act_r1.wig.gz
ChIPseq_ESC_Trim24adbkTaf3.PHDmutant_Flag_Act_r2.wig.gz
ChIPseq_ESC_Trim24adbkWT_IgG_Act_r1.wig.gz
ChIPseq_ESC_Trim24adbkWT_IgG_Act_r2.wig.gz
ChIPseq_HMEC_wt_p53_Unt_r1.wig.gz
ChIPseq_HMEC_wt_p53_Act_r1.wig.gz
ChIPseq_HMEC_wt_IgG_Unt_r1.wig.gz
ChIPseq_Neuron_dTrim24_p53_Act_r1.bw
ChIPseq_Neuron_dTrim24_p53_Act_r2.bw
ChIPseq_Neuron_dTrim24_Trim24_Act_r1.bw
ChIPseq_Neuron_dTrim24_Trim24_Act_r2.bw
ChIPseq_Neuron_dTrim24_IgG_Act_r1.bw
ChIPseq_Neuron_dTrim24_IgG_Act_r2.bw
ChIPseq_ESC_Trim24adbkRINGmutant_Flag_Act_r1.bw
ChIPseq_ESC_Trim24adbkRINGmutant_Flag_Act_r2.bw

Genome browser session
(e.g. UCSC)

The following files can be uploaded (all at once) to the UCSC genome browser by pasting all (mm10 or hg19) URLs into "Paste URLs or data" in "add custom tracks".
http://www.fmi.ch/groupdata/gschub/Trim24/ChIPseq_ESC_DNMT.TKO_p53_r1.bw
http://www.fmi.ch/groupdata/gschub/Trim24/ChIPseq_ESC_DNMT.TKO_p53_r2.bw
http://www.fmi.ch/groupdata/gschub/Trim24/ChIPseq_ESC_dp53_H3K27ac_Act_r1.bw
http://www.fmi.ch/groupdata/gschub/Trim24/ChIPseq_ESC_dp53_H3K27ac_Act_r2.bw
http://www.fmi.ch/groupdata/gschub/Trim24/ChIPseq_ESC_dp53_H3K27ac_Degr_r1.bw
http://www.fmi.ch/groupdata/gschub/Trim24/ChIPseq_ESC_dp53_H3K27ac_Degr_r2.bw
http://www.fmi.ch/groupdata/gschub/Trim24/ChIPseq_ESC_dp53_H3K27ac_Unt_r1.bw
http://www.fmi.ch/groupdata/gschub/Trim24/ChIPseq_ESC_dp53_H3K27ac_Unt_r2.bw
http://www.fmi.ch/groupdata/gschub/Trim24/ChIPseq_ESC_dp53_IgG_Unt_r1.bw
http://www.fmi.ch/groupdata/gschub/Trim24/ChIPseq_ESC_dp53_IgG_Unt_r2.bw
http://www.fmi.ch/groupdata/gschub/Trim24/ChIPseq_ESC_dp53_p53_Act_r1.bw
http://www.fmi.ch/groupdata/gschub/Trim24/ChIPseq_ESC_dp53_p53_Act_r2.bw
http://www.fmi.ch/groupdata/gschub/Trim24/ChIPseq_ESC_dp53_p53_Unt_r1.bw
http://www.fmi.ch/groupdata/gschub/Trim24/ChIPseq_ESC_dp53_p53_Unt_r2.bw
http://www.fmi.ch/groupdata/gschub/Trim24/ChIPseq_ESC_dp53_Trim24_Degr_r1.bw
http://www.fmi.ch/groupdata/gschub/Trim24/ChIPseq_ESC_dp53_Trim24_Degr_r2.bw
http://www.fmi.ch/groupdata/gschub/Trim24/ChIPseq_ESC_dp53_Trim24_Unt_r1.bw
http://www.fmi.ch/groupdata/gschub/Trim24/ChIPseq_ESC_dp53_Trim24_Unt_r2.bw
http://www.fmi.ch/groupdata/gschub/Trim24/ChIPseq_ESC_dTrim24_IgG_Unt_r1.bw
http://www.fmi.ch/groupdata/gschub/Trim24/ChIPseq_ESC_dTrim24_IgG_Unt_r2.bw
http://www.fmi.ch/groupdata/gschub/Trim24/ChIPseq_ESC_dTrim24_p53_Act_r1.bw
http://www.fmi.ch/groupdata/gschub/Trim24/ChIPseq_ESC_dTrim24_p53_Act_r2.bw
http://www.fmi.ch/groupdata/gschub/Trim24/ChIPseq_ESC_dTrim24_p53_DegrAct_r1.bw
http://www.fmi.ch/groupdata/gschub/Trim24/ChIPseq_ESC_dTrim24_p53_DegrAct_r2.bw
http://www.fmi.ch/groupdata/gschub/Trim24/ChIPseq_ESC_dTrim24_p53_Degr_r1.bw
http://www.fmi.ch/groupdata/gschub/Trim24/ChIPseq_ESC_dTrim24_p53_Degr_r2.bw
http://www.fmi.ch/groupdata/gschub/Trim24/ChIPseq_ESC_dTrim24_p53_Unt_r1.bw
http://www.fmi.ch/groupdata/gschub/Trim24/ChIPseq_ESC_dTrim24_p53_Unt_r2.bw
http://www.fmi.ch/groupdata/gschub/Trim24/ChIPseq_ESC_parental_p53_r1.bw
http://www.fmi.ch/groupdata/gschub/Trim24/ChIPseq_ESC_parental_p53_r2.bw
http://www.fmi.ch/groupdata/gschub/Trim24/ChIPseq_ESC_Trim24adbkING1.PHDmutant_Flag_Act_r1.bw
http://www.fmi.ch/groupdata/gschub/Trim24/ChIPseq_ESC_Trim24adbkING1.PHDmutant_Flag_Act_r2.bw
http://www.fmi.ch/groupdata/gschub/Trim24/ChIPseq_ESC_Trim24adbkNdeletion_Flag_Act_r1.bw
http://www.fmi.ch/groupdata/gschub/Trim24/ChIPseq_ESC_Trim24adbkNdeletion_Flag_Act_r2.bw
http://www.fmi.ch/groupdata/gschub/Trim24/ChIPseq_ESC_Trim24adbkNterminal_Flag_Act_r1.bw
http://www.fmi.ch/groupdata/gschub/Trim24/ChIPseq_ESC_Trim24adbkNterminal_Flag_Act_r2.bw
http://www.fmi.ch/groupdata/gschub/Trim24/ChIPseq_ESC_Trim24adbkPHDmutant_Flag_Act_r1.bw
http://www.fmi.ch/groupdata/gschub/Trim24/ChIPseq_ESC_Trim24adbkPHDmutant_Flag_Act_r2.bw
http://www.fmi.ch/groupdata/gschub/Trim24/ChIPseq_ESC_Trim24adbkTaf3.PHDmutant_Flag_Act_r1.bw
http://www.fmi.ch/groupdata/gschub/Trim24/ChIPseq_ESC_Trim24adbkTaf3.PHDmutant_Flag_Act_r2.bw
http://www.fmi.ch/groupdata/gschub/Trim24/ChIPseq_ESC_Trim24adbkWT_Flag_Act_r1.bw
http://www.fmi.ch/groupdata/gschub/Trim24/ChIPseq_ESC_Trim24adbkWT_Flag_Act_r2.bw
http://www.fmi.ch/groupdata/gschub/Trim24/ChIPseq_ESC_Trim24adbkWT_IgG_Act_r1.bw
http://www.fmi.ch/groupdata/gschub/Trim24/ChIPseq_ESC_Trim24adbkWT_IgG_Act_r2.bw
http://www.fmi.ch/groupdata/gschub/Trim24/ChIPseq_ESC_wt_H3K23ac_Unt_r1.bw
http://www.fmi.ch/groupdata/gschub/Trim24/ChIPseq_ESC_wt_H3K23ac_Unt_r2.bw
http://www.fmi.ch/groupdata/gschub/Trim24/ChIPseq_ESC_wt_IgG_Unt_r1.bw
http://www.fmi.ch/groupdata/gschub/Trim24/ChIPseq_ESC_wt_IgG_Unt_r2.bw
http://www.fmi.ch/groupdata/gschub/Trim24/ChIPseq_ESC_wt_p53_Act_r1.bw
http://www.fmi.ch/groupdata/gschub/Trim24/ChIPseq_ESC_wt_p53_Act_r2.bw
http://www.fmi.ch/groupdata/gschub/Trim24/ChIPseq_ESC_wt_p53_Unt_r1.bw
http://www.fmi.ch/groupdata/gschub/Trim24/ChIPseq_ESC_wt_p53_Unt_r2.bw
http://www.fmi.ch/groupdata/gschub/Trim24/ChIPseq_ESC_wt_Trim24_Act_r1.bw
http://www.fmi.ch/groupdata/gschub/Trim24/ChIPseq_ESC_wt_Trim24_Act_r2.bw
http://www.fmi.ch/groupdata/gschub/Trim24/ChIPseq_ESC_wt_Trim24_Unt_r1.bw
http://www.fmi.ch/groupdata/gschub/Trim24/ChIPseq_ESC_wt_Trim24_Unt_r2.bw
http://www.fmi.ch/groupdata/gschub/Trim24/ChIPseq_HMEC_wt_IgG_Unt_r1.bw
http://www.fmi.ch/groupdata/gschub/Trim24/ChIPseq_HMEC_wt_p53_Act_r1.bw
http://www.fmi.ch/groupdata/gschub/Trim24/ChIPseq_HMEC_wt_p53_Unt_r1.bw
http://www.fmi.ch/groupdata/gschub/Trim24/ChIPseq_ESC_Trim24adbkRINGmutant_Flag_Act_r1.bw
http://www.fmi.ch/groupdata/gschub/Trim24/ChIPseq_ESC_Trim24adbkRINGmutant_Flag_Act_r2.bw
http://www.fmi.ch/groupdata/gschub/Trim24/ChIPseq_Neuron_dTrim24_IgG_Act_r1.bw
http://www.fmi.ch/groupdata/gschub/Trim24/ChIPseq_Neuron_dTrim24_IgG_Act_r2.bw
http://www.fmi.ch/groupdata/gschub/Trim24/ChIPseq_Neuron_dTrim24_p53_Act_r1.bw
http://www.fmi.ch/groupdata/gschub/Trim24/ChIPseq_Neuron_dTrim24_p53_Act_r2.bw
http://www.fmi.ch/groupdata/gschub/Trim24/ChIPseq_Neuron_dTrim24_Trim24_Act_r1.bw
http://www.fmi.ch/groupdata/gschub/Trim24/ChIPseq_Neuron_dTrim24_Trim24_Act_r2.bw

Methodology

Replicates

Between 1 and 3 biological replicates were performed per cell type and antibody (indicated).

Sequencing depth

All ChIP-seq samples with total and uniquely mapped reads are given below, fields separated by a whitespace character):
"Sample Name" "Total Reads" "Number of Mapped Reads" "(% mapped of total reads)"
ChIPseq_ESC_parental_p53_r2_1 12,953,208 9,138,025 (70.55%)
ChIPseq_ESC_parental_p53_r2_2 12,868,467 9,079,786 (70.56%)
ChIPseq_ESC_parental_p53_r2_3 12,837,028 9,058,520 (70.57%)
ChIPseq_ESC_parental_p53_r1_1 9,905,005 6,918,496 (69.85%)
ChIPseq_ESC_parental_p53_r1_2 9,835,309 6,873,050 (69.88%)
ChIPseq_ESC_parental_p53_r1_3 9,810,865 6,851,888 (69.84%)
ChIPseq_ESC_DNMT-TKO_p53_r2_1 6,770,528 4,574,583 (67.57%)
ChIPseq_ESC_DNMT-TKO_p53_r2_2 6,719,088 4,540,375 (67.57%)
ChIPseq_ESC_DNMT-TKO_p53_r2_3 6,695,131 4,526,319 (67.61%)
ChIPseq_ESC_DNMT-TKO_p53_r1_1 18,932,477 13,311,092 (70.31%)
ChIPseq_ESC_DNMT-TKO_p53_r1_2 18,803,122 13,224,555 (70.33%)
ChIPseq_ESC_DNMT-TKO_p53_r1_3 18,749,302 13,180,603 (70.3%)
ChIPseq_ESC_dp53_p53_Unt_r1_1 65,650,470 45,851,588 (69.84%)
ChIPseq_ESC_dp53_p53_Act_r1_1 54,870,442 35,350,934 (64.43%)
ChIPseq_ESC_dp53_p53_Unt_r2_1 30,098,786 18,505,214 (61.48%)
ChIPseq_ESC_dp53_p53_Act_r2_1 25,294,318 14,013,762 (55.4%)
ChIPseq_ESC_dp53_IgG_Unt_r1_1 72,435,814 49,401,636 (68.2%)
ChIPseq_ESC_dp53_IgG_Unt_r2_1 20,559,738 11,895,862 (57.86%)
ChIPseq_ESC_dp53_H3K27ac_Degr_r1_1 24,878,288 18,319,770 (73.64%)
ChIPseq_ESC_dp53_H3K27ac_Degr_r1_2 34,066,676 25,226,852 (74.05%)
ChIPseq_ESC_dp53_H3K27ac_Degr_r2_1 22,354,858 16,732,718 (74.85%)
ChIPseq_ESC_dp53_H3K27ac_Degr_r2_2 30,693,712 23,081,518 (75.2%)
ChIPseq_ESC_dp53_H3K27ac_Unt_r1_1 24,178,504 18,384,046 (76.03%)
ChIPseq_ESC_dp53_H3K27ac_Unt_r1_2 32,110,052 24,484,888 (76.25%)
ChIPseq_ESC_dp53_H3K27ac_Unt_r2_1 24,369,268 18,232,332 (74.82%)
ChIPseq_ESC_dp53_H3K27ac_Unt_r2_2 33,483,146 25,164,086 (75.15%)
ChIPseq_ESC_dp53_H3K27ac_Act_r1_1 20,451,674 15,889,030 (77.69%)
ChIPseq_ESC_dp53_H3K27ac_Act_r1_2 28,186,454 21,935,662 (77.82%)
ChIPseq_ESC_dp53_H3K27ac_Act_r2_1 27,526,396 21,121,196 (76.73%)
ChIPseq_ESC_dp53_H3K27ac_Act_r2_2 37,892,840 29,095,296 (76.78%)
ChIPseq_ESC_dp53_Trim24_Degr_r1_1 16,457,611 11,534,776 (70.09%)
ChIPseq_ESC_dp53_Trim24_Degr_r1_2 16,525,719 11,587,707 (70.12%)
ChIPseq_ESC_dp53_Trim24_Degr_r1_3 17,313,782 12,102,110 (69.9%)
ChIPseq_ESC_dp53_Trim24_Degr_r2_1 11,545,730 8,167,792 (70.74%)
ChIPseq_ESC_dp53_Trim24_Degr_r2_2 11,587,046 8,214,139 (70.89%)
ChIPseq_ESC_dp53_Trim24_Degr_r2_3 12,172,483 8,575,628 (70.45%)
ChIPseq_ESC_dp53_Trim24_Unt_r1_1 14,246,311 9,839,182 (69.06%)
ChIPseq_ESC_dp53_Trim24_Unt_r1_2 14,344,744 9,928,385 (69.21%)
ChIPseq_ESC_dp53_Trim24_Unt_r1_3 14,957,362 10,282,743 (68.75%)
ChIPseq_ESC_dp53_Trim24_Unt_r2_1 11,402,107 8,007,135 (70.23%)
ChIPseq_ESC_dp53_Trim24_Unt_r2_2 11,436,004 8,040,556 (70.31%)
ChIPseq_ESC_dp53_Trim24_Unt_r2_3 12,098,122 8,470,457 (70.01%)
ChIPseq_ESC_wt_H3K23ac_Unt_r1_1 25,765,492 11,263,948 (43.72%)
ChIPseq_ESC_wt_H3K23ac_Unt_r1_2 25,264,816 11,041,062 (43.7%)
ChIPseq_ESC_wt_H3K23ac_Unt_r2_1 29,734,195 20,747,167 (69.78%)
ChIPseq_ESC_wt_H3K23ac_Unt_r2_2 29,223,281 20,368,672 (69.7%)
ChIPseq_ESC_Trim24adbkWT_Flag_Act_r1_1 34,297,644 23,887,316 (69.65%)
ChIPseq_ESC_Trim24adbkWT_Flag_Act_r2_1 41,450,126 29,686,342 (71.62%)
ChIPseq_ESC_Trim24adbkWT_IgG_Act_r1_1 27,364,335 15,869,653 (57.99%)
ChIPseq_ESC_Trim24adbkPHDmutant_Flag_Act_r1_1 41,703,436 24,836,233 (59.55%)
ChIPseq_ESC_Trim24adbkPHDmutant_Flag_Act_r2_1 42,933,211 29,505,696 (68.72%)
ChIPseq_ESC_Trim24adbkWT_IgG_Act_r2_1 24,183,232 10,527,173 (43.53%)
ChIPseq_ESC_Trim24adbkWT_IgG_Act_r2_2 23,481,859 10,443,979 (44.48%)
ChIPseq_ESC_Trim24adbkNterminal_Flag_Act_r1_1 21,926,675 10,178,764 (46.42%)
ChIPseq_ESC_Trim24adbkNterminal_Flag_Act_r1_2 21,443,673 10,103,900 (47.12%)
ChIPseq_ESC_Trim24adbkNterminal_Flag_Act_r2_1 25,029,186 11,038,186 (44.1%)
ChIPseq_ESC_Trim24adbkNterminal_Flag_Act_r2_2 24,295,945 10,898,500 (44.86%)
ChIPseq_ESC_Trim24adbkNdeletion_Flag_Act_r1_1 20,627,237 8,890,686 (43.1%)
ChIPseq_ESC_Trim24adbkNdeletion_Flag_Act_r1_2 20,031,511 8,846,018 (44.16%)
ChIPseq_ESC_Trim24adbkNdeletion_Flag_Act_r2_1 21,401,368 5,834,178 (27.26%)
ChIPseq_ESC_Trim24adbkNdeletion_Flag_Act_r2_2 20,595,848 5,787,512 (28.1%)
ChIPseq_ESC_Trim24adbkING1-PHDmutant_Flag_Act_r1_1 22,861,941 10,186,580 (44.56%)
ChIPseq_ESC_Trim24adbkING1-PHDmutant_Flag_Act_r1_2 22,116,266 10,068,778 (45.53%)
ChIPseq_ESC_Trim24adbkING1-PHDmutant_Flag_Act_r2_1 20,838,444 8,153,291 (39.13%)
ChIPseq_ESC_Trim24adbkING1-PHDmutant_Flag_Act_r2_2 20,297,997 8,105,359 (39.93%)
ChIPseq_ESC_Trim24adbkTaf3-PHDmutant_Flag_Act_r1_1 30,447,481 21,997,548 (72.25%)
ChIPseq_ESC_Trim24adbkTaf3-PHDmutant_Flag_Act_r1_2 30,312,481 21,904,070 (72.26%)
ChIPseq_ESC_Trim24adbkTaf3-PHDmutant_Flag_Act_r2_1 25,447,628 14,569,018 (57.25%)
ChIPseq_ESC_Trim24adbkTaf3-PHDmutant_Flag_Act_r2_2 25,005,728 14,492,250 (57.96%)
ChIPseq_ESC_dTrim24_IgG_Unt_r2_1 14,345,921 10,221,775 (71.25%)
ChIPseq_ESC_dTrim24_IgG_Unt_r2_2 14,284,836 10,177,088 (71.24%)
ChIPseq_ESC_wt_p53_Unt_r1_1 18,277,396 13,181,572 (72.12%)
ChIPseq_ESC_wt_p53_Unt_r1_2 18,214,624 13,138,640 (72.13%)

ChIPseq_ESC_wt_p53_Unt_r2_1 19,237,225 13,947,011 (72.5%)
ChIPseq_ESC_wt_p53_Unt_r2_2 19,165,441 13,895,364 (72.5%)
ChIPseq_ESC_wt_Trim24_Unt_r1_1 13,220,042 9,245,396 (69.93%)
ChIPseq_ESC_wt_Trim24_Unt_r1_2 13,177,635 9,216,437 (69.94%)
ChIPseq_ESC_wt_Trim24_Unt_r2_1 13,787,950 9,650,196 (69.99%)
ChIPseq_ESC_wt_Trim24_Unt_r2_2 13,753,655 9,623,863 (69.97%)
ChIPseq_ESC_wt_p53_Act_r1_1 15,576,820 11,639,504 (74.72%)
ChIPseq_ESC_wt_p53_Act_r1_2 15,515,125 11,590,100 (74.7%)
ChIPseq_ESC_wt_p53_Act_r2_1 14,229,708 10,680,562 (75.06%)
ChIPseq_ESC_wt_p53_Act_r2_2 14,187,697 10,652,432 (75.08%)
ChIPseq_ESC_wt_Trim24_Act_r1_1 15,838,495 11,594,358 (73.2%)
ChIPseq_ESC_wt_Trim24_Act_r1_2 15,801,167 11,565,553 (73.19%)
ChIPseq_ESC_wt_Trim24_Act_r2_1 17,875,401 13,087,161 (73.21%)
ChIPseq_ESC_wt_Trim24_Act_r2_2 17,813,960 13,042,136 (73.21%)
ChIPseq_ESC_wt_IgG_Unt_r1_1 18,811,200 13,242,154 (70.4%)
ChIPseq_ESC_wt_IgG_Unt_r1_2 18,743,881 13,197,789 (70.41%)
ChIPseq_ESC_wt_IgG_Unt_r2_1 19,496,293 13,726,192 (70.4%)
ChIPseq_ESC_wt_IgG_Unt_r2_2 19,416,802 13,667,710 (70.39%)
ChIPseq_ESC_dTrim24_p53_Act_r1_1 14,636,795 10,938,873 (74.74%)
ChIPseq_ESC_dTrim24_p53_Act_r1_2 14,581,104 10,896,005 (74.73%)
ChIPseq_ESC_dTrim24_p53_Act_r2_1 16,577,663 12,375,827 (74.65%)
ChIPseq_ESC_dTrim24_p53_Act_r2_2 16,513,287 12,330,992 (74.67%)
ChIPseq_ESC_dTrim24_p53_DegrAct_r1_1 17,989,487 13,459,029 (74.82%)
ChIPseq_ESC_dTrim24_p53_DegrAct_r1_2 17,942,976 13,423,519 (74.81%)
ChIPseq_ESC_dTrim24_p53_DegrAct_r2_1 16,984,313 12,729,584 (74.95%)
ChIPseq_ESC_dTrim24_p53_DegrAct_r2_2 16,932,101 12,693,697 (74.97%)
ChIPseq_ESC_dTrim24_IgG_Unt_r1_1 14,272,462 10,166,193 (71.23%)
ChIPseq_ESC_dTrim24_IgG_Unt_r1_2 14,227,695 10,133,986 (71.23%)
ChIPseq_ESC_dTrim24_p53_Degr_r1_1 20,904,129 19,167,812 (91.69%)
ChIPseq_ESC_dTrim24_p53_Degr_r1_2 21,019,639 19,265,267 (91.65%)
ChIPseq_HMEC_wt_IgG_Unt_r1_1 21,799,136 20,713,143 (95.02%)
ChIPseq_HMEC_wt_IgG_Unt_r1_2 21,859,376 20,774,965 (95.04%)
ChIPseq_HMEC_wt_p53_Act_r1_1 18,362,762 17,366,226 (94.57%)
ChIPseq_HMEC_wt_p53_Act_r1_2 18,454,784 17,444,492 (94.53%)
ChIPseq_ESC_dTrim24_p53_Degr_r2_1 19,954,676 18,151,799 (90.97%)
ChIPseq_ESC_dTrim24_p53_Degr_r2_2 20,043,439 18,230,632 (90.96%)
ChIPseq_ESC_dTrim24_p53_Unt_r1_1 19,056,114 17,480,096 (91.73%)
ChIPseq_ESC_dTrim24_p53_Unt_r1_2 19,149,842 17,559,560 (91.7%)
ChIPseq_ESC_dTrim24_p53_Unt_r2_1 20,978,464 19,354,695 (92.26%)
ChIPseq_ESC_dTrim24_p53_Unt_r2_2 21,091,983 19,448,119 (92.21%)
ChIPseq_HMEC_wt_p53_Unt_r1_1 19,663,003 18,424,889 (93.7%)
ChIPseq_HMEC_wt_p53_Unt_r1_2 19,768,646 18,515,755 (93.66%)

Antibodies

p53-2524-Cell signaling technology, Flag-F1804-Sigma Aldrich, Trim24-A300-815A-Bethyl laboratories, IgG-M7023-Sigma Aldrich, H3K23ac-39131-Active Motif, H3K27ac-39133-Active Motif

Peak calling parameters

Peak calling on all datasets were performed with MACS297 (version 2.1.3.3) using the callpeak argument with default settings and specifying the genome size with -g mm or -hs for mouse or human, respectively. Peaks were called for mouse ChIPseq datasets using matched IgG ChIPseq datasets as controls. Peaks were called for human ChIPseq datasets using matched IgG ChIPseq.

Data quality

ChIP-seq sample quality was assessed using the following criteria:
- technical quality (sufficient sequencing depth and unique-hit mapping rates)
- reproducibility (high Pearsons's correlation coefficient on the level of peaks).
-Quality control of sequencing data was carried out by Qualimap127 (v.2.2.1) using ‘bamqc’ and ‘rnaseq’ modes. The quality of ChIPseq, as well as ATACseq and RNAseq datasets were further assessed by R ChIPQC128 package (v1.28.0) (indicated in manuscript).

Software

Software used for data collection: For ChIPseq, Illumina RTA 1.18.64 and bcl2fastq2 v2.17 was used for basecalling and demultiplexing for single-read experiments, Illumina RTA 2.4.11 and bcl2fastq2 v2.17 was used for basecalling and demultiplexing for paired-read experiments.

Software used for data analysis: MACS2 peak caller (version 2.1.3.3), bedtools (version 2.25.0), Homer motif finder (version 4.8). Data analysis was performed in R (4.2) and using BSgenome.Mmusculus.UCSC.mm10 1.4.0, BSgenome.Hsapiens.UCSC.hg38 1.4.1, TxDb.Mmusculus.UCSC.mm10.knownGene 3.4.4, QuasR 1.26.0 using Rbowtie 1.26.0, Gviz 1.40.1, ChIPseeker 1.28.3, Biostrings 2.54.0, GenomicRanges 1.48.0.
