# Supplementary figures and images for: Readout of histone methylation by Trim24 locally restricts chromatin opening by p53
Source: Nat Struct Mol Biol. 2023 Jun 29;30(7):948–57. doi: 10.1038/s41594-023-01021-8 (PMC10352137; doi:10.1038/s41594-023-01021-8)

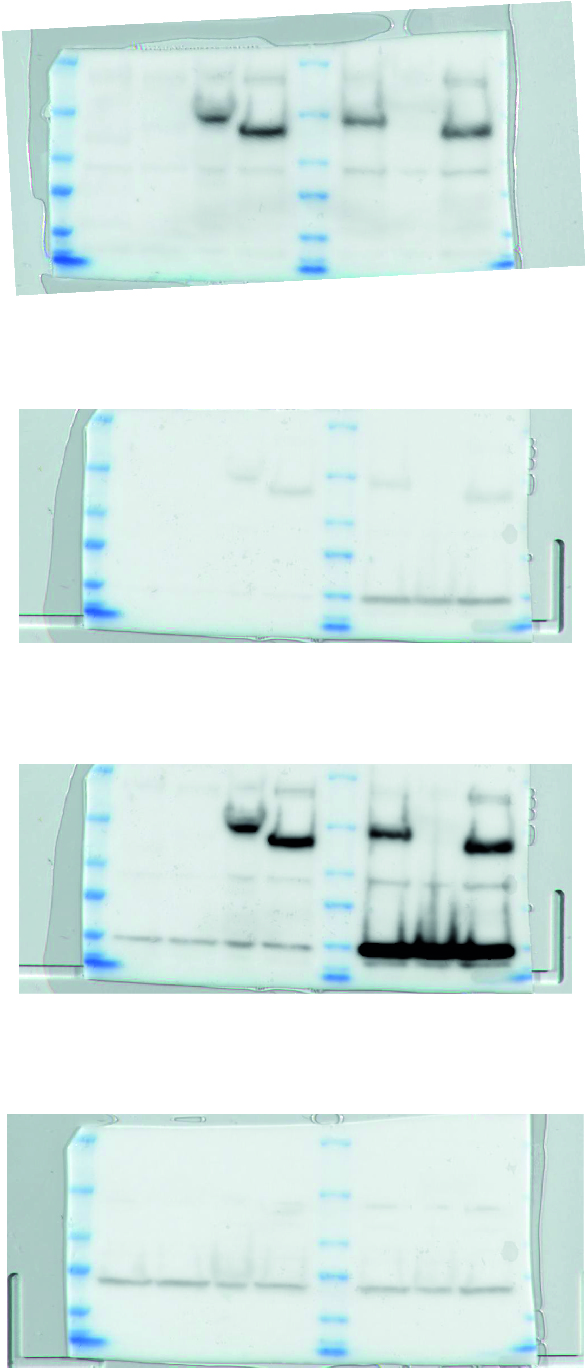

Supplement: Source Data for Fig. 2 — Raw western blot images for Fig. 2. [file 41594_2023_1021_MOESM6_ESM.jpg]

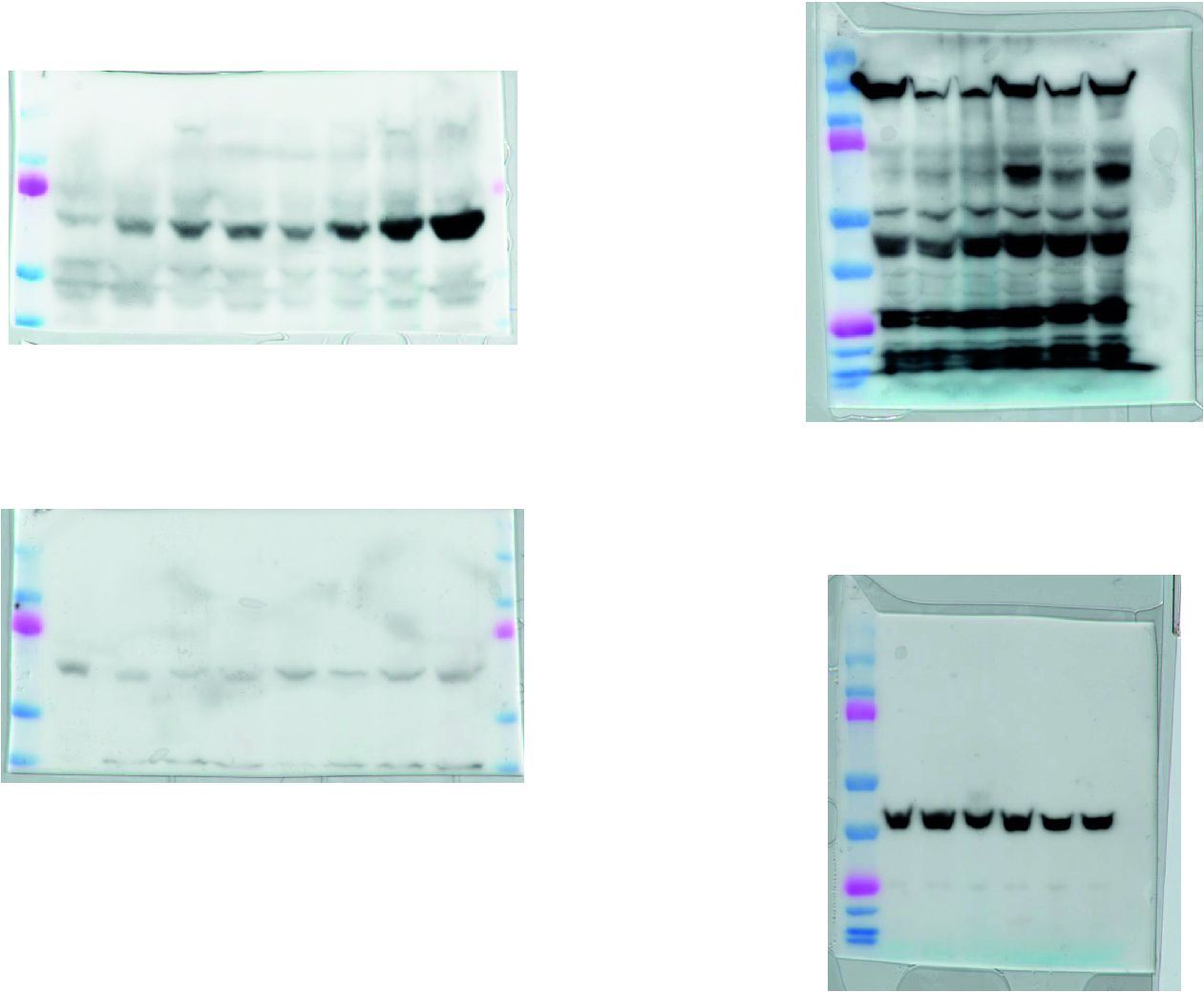

Supplement: Source Data for Fig. 4 — Raw western blot images for Fig. 4. [file 41594_2023_1021_MOESM7_ESM.jpg]
